# The Role of Aryldiazonium Chemistry in Designing Electrochemical Aptasensors for the Detection of Food Contaminants

**DOI:** 10.3390/ma14143857

**Published:** 2021-07-10

**Authors:** Matei Raicopol, Luisa Pilan

**Affiliations:** 1 Costin Nenitzescu, Department of Organic Chemistry, Faculty of Applied Chemistry and Material Science, University Politehnica of Bucharest, 1-7 Gheorghe Polizu, 011061 Bucharest, Romania; m_raicopol@chim.upb.ro; 2 Department of Inorganic Chemistry, Physical Chemistry and Electrochemistry, Faculty of Applied Chemistry and Material Science, University Politehnica of Bucharest, 1-7 Gheorghe Polizu, 011061 Bucharest, Romania

**Keywords:** aryldiazonium chemistry, aptasensor, electrochemical detection, food contaminants

## Abstract

Food safety monitoring assays based on synthetic recognition structures such as aptamers are receiving considerable attention due to their remarkable advantages in terms of their ability to bind to a wide range of target analytes, strong binding affinity, facile manufacturing, and cost-effectiveness. Although aptasensors for food monitoring are still in the development stage, the use of an electrochemical detection route, combined with the wide range of materials available as transducers and the proper immobilization strategy of the aptamer at the transducer surface, can lead to powerful analytical tools. In such a context, employing aryldiazonium salts for the surface derivatization of transducer electrodes serves as a simple, versatile and robust strategy to fine-tune the interface properties and to facilitate the convenient anchoring and stability of the aptamer. By summarizing the most important results disclosed in the last years, this article provides a comprehensive review that emphasizes the contribution of aryldiazonium chemistry in developing electrochemical aptasensors for food safety monitoring.

## 1. Introduction

Ensuring the safety and quality of food is a global problem that deserves great attention. According to the World Health Organization, “an estimated 600 million—almost 1 in 10 people in the world—fall ill after eating contaminated food and 420,000 die every year” [1]. Food safety is often affected by factors like the presence of antibiotics, pesticides, pathogens, toxins, heavy metals, and other toxic substances that enter the food chain through different pathways [2,3,4]. Foodborne diseases vary in severity, from temporary discomfort to serious illness such as cardiovascular or neurological disorders, increased cancer risk, etc. [5,6]. Moreover, the extensive use of antibiotics in animal feed in order to increase production is associated with the spread of resistant bacterial strains for which current treatments are ineffective [1]. Despite their high sensitivity, many of the traditional assays employed for detection of food contaminants, such as HPLC and mass spectrometry, are expensive, laborious, and demand highly trained staff, and therefore have limited suitability for areas with poorly equipped facilities and shortages of specialists [7,8,9,10,11,12]. In this context, the efforts to develop accurate, easy-to-use, disposable, and inexpensive devices for food safety control have greatly expanded. Biosensor technology is able to complement or even replace established analytical methods, by simplifying the sample preparation steps and reducing the analysis time and costs. Electrochemical biosensors are devices that combine the sensitivity of electroanalytical methods and the intrinsic selectivity of biomolecules, with additional benefits such as low cost, ease of fabrication, feasibility for miniaturization, and multiplexed detection [13]. Moreover, the use of nucleic acid aptamers as biorecognition elements with extremely high binding affinity to a variety of targets, ranging from small molecules to bacteria, has broadened the range of biosensor applications [14,15]. Nucleic acid aptamers are single-stranded DNA (ssDNA) or RNA sequences isolated from a vast library of synthetic random oligonucleotides via an automated process known as Systematic Evolution of Ligands by Exponential Enrichment (SELEX) [16]. Aptamer molecules can compete with antibodies in terms of affinity and specificity, have superior chemical stability, and, unlike antibodies, can be reversibly denatured in order to release the target analyte and enable device reusability [17,18,19]. As aptamers can be obtained through custom synthesis at reasonable prices, they are increasingly replacing antibodies in the fabrication of affinity biosensors [20]. The scientific literature provides numerous examples of aptasensors with suitable sensitivity, specificity, and selectivity to be used for detecting a wide range of food contaminants, such as veterinary drugs, pesticides, preservatives, packaging components, seafood toxins, allergens, heavy metals, bacteria, etc. [2,21,22,23,24,25,26,27,28,29]. As more aptamers are being developed and become commercially available, the research in this area is anticipated to show important growth [30].

Although the analytical performance of biosensors in terms of selectivity, sensitivity, and stability is mainly governed by intrinsic bioreceptor characteristics [31], many other factors that affect device performance can be regulated by the strategy employed for biomolecule immobilization [32]. The choice of the immobilization technique may affect a wide range of parameters, including the preservation of biological activity, the accessibility and stability of surface-confined receptor molecules, the control of non-specific adsorption, the response time, the sensitivity, and the overall stability of the biosensor [32]. Hence, the selection of a robust derivatization strategy for the transducer surface is a prerequisite in adjusting the interface properties and promoting a convenient immobilization of the aptamer [33,34,35,36]. In this context, aryldiazonium chemistry has emerged as a powerful and versatile modification procedure that allows the tailoring of the chemical and electronic properties of the sensing platform and enables proper distribution, surface density, and stability for the aptamer contained in the recognition layer [34,36,37]. Furthermore, by taking advantage of the electro-addressability of the electrochemical grafting route, concurrent coupling of different aptamer bioreceptors has been demonstrated, which allowed the simultaneous detection of different target analytes [38]. This is particularly important as the development of accurate and sensitive sensing devices performing simultaneous multiplex detection represents a current challenge for food safety screening [39]. Moreover, the concomitant grafting of functionalities with an antifouling role, aimed at reducing nonspecific interactions with interfering compounds, proved to increase the selectivity of the assay without affecting the simplicity of the approach [35,40,41].

The first part of this review provides an overview of the role that aptamers play as biorecognition elements for biosensors, briefly explores the most common aptasensing strategies, and evaluates their shortcomings. Thereon, the basic concepts and challenges involved in aryldiazonium grafting are outlined by emphasizing the role of this particular modification approach in developing aptasensing assays intended for applications related to the most commonly encountered food contaminants. An updated overview that focuses on relevant examples of electrochemical aptasensors developed via aryldiazonium grafting, classified according to the type of food contaminant, is further provided.

## 2. The Role of Aptamers in Developing Biosensors for Food Contaminants

One of the most important advantages of electrochemical aptasensors as analytical tools for the assessment of food quality and safety is their suitability for detecting a large variety of chemical substances. Aptamers can be produced for a wide range of targets, from small molecules like antibiotics, pesticides, herbicides, and toxins up to proteins and even bacteria [42,43]. Moreover, aptamers serve as very versatile biorecognition molecules for biosensors because of their straightforward chemical modification with functional groups that enable surface immobilization or act as signaling elements (i.e., electrochemical and fluorescent probes). Also, they undergo conformational changes upon target binding (G-quaduplex, hairpin) [44,45], which facilitate the ultrasensitive detection of analytes. Aptamers are regarded as a promising alternative to antibodies, as they possess a high affinity and specificity for a wider spectrum of targets, a higher stability at elevated temperatures, can be thermally denaturated reversibly, and their synthesis is straightforward and cost-effective [45]. Nowadays, DNA aptamers are preferred over their RNA analogues as they possess several important advantages, mainly relating to their chemical and biological stability [46,47].

The binding events occurring between aptamers and their target molecules usually lead to conformational changes of the oligonucleotide chains, which are folded into specific secondary and tertiary structures [48]. It was reported that the selective recognition of targets is determined by their interaction with nucleotides via hydrogen bonding, π-stacking, van der Waals and electrostatic forces [46,48,49]. The strength of the interaction leading to aptamer–target complexes is defined by the dissociation constant, K_d_, which can extend from the micromolar domain (low affinity) to the picomolar domain (high affinity) [41,50].

Currently, one of the most important challenges in the design of aptasensors is to obtain new aptamers possessing excellent binding affinity for the envisaged target analyte. Aptamers are commonly generated through an iterative in vitro selection and amplification protocol called SELEX. The originally developed SELEX method requires the immobilization of targets on a suitable matrix, followed by the addition of free oligonucleotide libraries [51]. Basically, a typical SELEX cycle consists of successive steps that comprise target binding, washing to remove unbound oligonucleotides, enzymatic amplification of target-bound oligonucleotides, and purification of the selected pool of oligonucleotides for the next selection cycle [52]. The oligonucleotides selected and enriched during several SELEX cycles (5–15) are further sequenced and cloned in order to isolate the sequence that is highly specific for a certain target, which can be ultimately employed as biosensor receptor [53]. While the originally reported SELEX protocol is very laborious, variations on the method have since been reported that attempted to increase the yield and time efficiency of the selection process or generate aptamers with higher affinities [47]. Currently, the time necessary for developing an aptamer has decreased from months (required for conventional SELEX) to several hours. Furthermore, accurate in silico methods for aptamer selection are able to model and predict aptamer–target interactions without the need for physically conducting a SELEX process [47,54]. Although in silico selections are usually confirmed in vitro, they proved quite useful for improving the affinity of aptamers or ensuring that a particular conformation is stable [55]. Aptamer sequence truncation is an accepted practice that consists in removing the primer binding sites from the oligonucleotide sequence, which decreases the cost for aptamer synthesis and facilitates aptamer–target interaction studies [56]. Although some studies have suggested that shorter versions of aptamers display comparable affinity and specificity [57,58], a thorough analysis of the truncated aptamers’ secondary structures is recommended, which can be accomplished using several computational programs [56].

For the design of aptamers specific for food contaminants, a special variant of the SELEX technology, Capture-SELEX, is widely used [51]. This SELEX variant was developed by Stoltenburg et al. for the selection of DNA aptamers that bind to small target molecules that are not suitable for immobilization on solid surfaces [52]. In the Capture-SELEX process, the oligonucleotide library is immobilized on magnetic beads by hybridization with complementary (i.e., capture) oligonucleotides, and the aptamers are specifically eluted from the beads by adding a solution containing the target molecule [52]. Shortcomings of the Capture-SELEX method for aptamer screening have also been reported, such as “false positive” sequences that dissociate because they are weakly bound with the capture oligonucleotides or sequences that bind irreversibly to the support when target binding is not accompanied by suitable conformational changes [51].

The aptamer sequences obtained through SELEX technology that have been employed in the fabrication of electrochemical aptasensors for food contaminants and are further discussed in this manuscript are provided in Table 1.

## 3. Strategies in Electrochemical Aptasensor Design: Aptamer Immobilization and Electrochemical Signal Generation

### 3.1. Strategies for Aptamer Modification and Immobilization at the Electrode Substrate

Oligonucleotide aptamers can be easily modified by convenient functional groups like amino, azido, thiol, or biotin, which can be incorporated at the 3′ and/or 5′ ends during synthesis, in order to facilitate their labeling or immobilization onto different platforms. Several studies indicated that the 3′-end is more suitable for surface immobilization, as it may confer resistance to exonucleases [19,90]. Nevertheless, the 5′-end is frequently used for attachment, considering that modifications at this position are performed at the final stage of oligonucleotide synthesis, which improves yield and simplifies purification [19]. The additional incorporation of spacer groups between the oligonucleotide sequence and the attachment point can improve the aptamers’ conformational flexibility, ultimately leading to an enhanced sensitivity [19,76,91,92]. Several spacers, such as alkyl chains, polyethylene glycol (PEG), hexamethyldiamine (HMDA), or oligothymidine sequences (of all the DNA bases, thymidine displays the lowest nonspecific binding), proved to facilitate analyte recognition and binding [19,76,91,92]. Still, it should be borne in mind that a spacer group can potentially alter the aptamer’s secondary structure and, therefore, influence its affinity for the target molecule [19].

Besides the use of appropriate spacer groups in order to increase the distance between the aptamer binding site and the electrode surface, the aptamer packing density has a large influence on target binding [93]. Plaxco and coworkers investigated the effect of aptamer packing density on the performance of electrochemical aptasensors for different targets, comprising both small (i.e., cocaine) and large molecules (i.e., thrombin) [94]. For the cocaine sensor, the sensitivity diminished when the aptamers were densely packed, due to the occurrence of unfavorable interactions between neighboring aptamers (e.g., cross-hybridization). For the thrombin sensor, an optimal sensitivity was obtained at intermediate packing densities, which was explained by a decrease in target affinity due to crowding at higher densities, and an unfavorable raise in the mobility of the aptamer’s redox tag at lower packing densities [94].

**Scheme 1:**
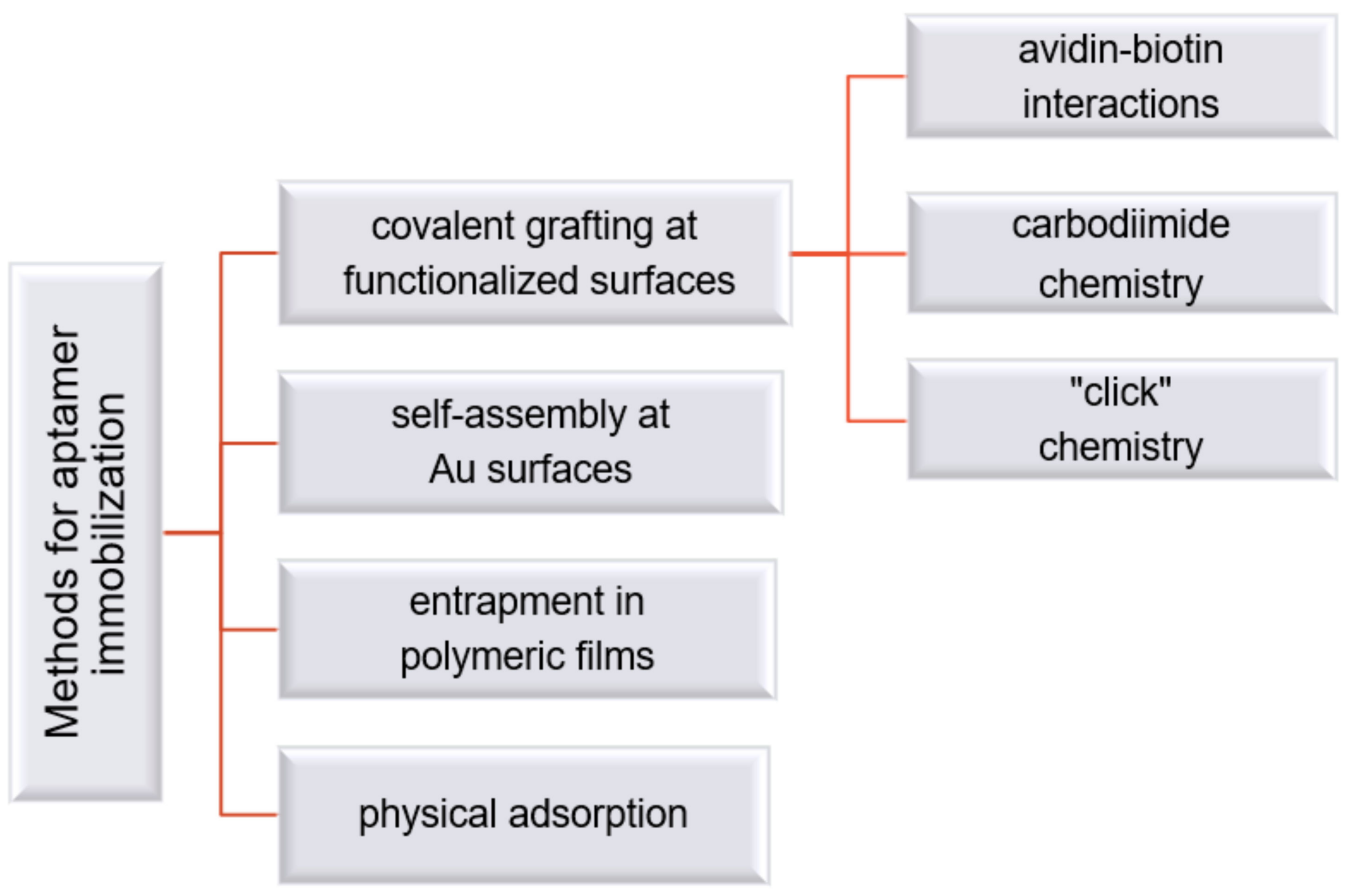
Different approaches reported for the aptamer immobilization for developing electrochemical aptasensors for food safety monitoring.

Both gold- and carbon-based electrodes are commonly employed as substrates for developing electrochemical aptasensors for food safety monitoring [42]. Different approaches have been reported for the immobilization of aptamers, such as physical adsorption [95,96], self-assembly [24,97,98], entrapment in polymer films [99], and covalent grafting at functionalized surfaces [61,100,101] (Scheme 1).

Although aptamers are generally stable even under nonphysiologically conditions, and can be easily modified through chemical reactions, the strategy adopted for surface immobilization plays a key role in preserving their target specificity [99,102]. For example, according to Vasilescu and Marty [42], aptamers immobilized using common approaches, such as self-assembly of thiolated aptamers on Au, assembly based on avidin–biotin interactions, and carbodiimide and “click” coupling, retained the same binding affinity as in solution. Moreover, procedures that afford a controlled functionalization of the electrode surface allow the immobilization of the optimal amount of aptamer in order to obtain the required packing density, besides ensuring an adequate stability of the sensing interface. In this context, the electrochemical reduction of aryldiazonium salts proved to be very attractive in terms of speed, simplicity, and the long term stability of the resulting layers [36]. An overview of modification procedures based on aryldiazonium chemistry is provided in Section 4 of this article.

### 3.2. Strategies for Aptamer Modification and Immobilization at the Electrode Substrate

The translation of the recognition event into an electrochemical signal can be achieved through both label-free and labeled detection strategies, where labeling involves either covalent or noncovalent binding. Generally, these strategies exploit differences in the flexibility of ssDNA and double-stranded DNA (dsDNA) structures, along with their transition towards secondary structures such as stem and loop structures, and are known as electrochemical molecular beacons [46,103], in analogy with previously developed fluorescent molecular beacons [104]. These approaches have been reviewed in several papers that highlight possible detection mechanisms for electrochemical aptasensors [41,42,45,50,90,105,106,107]. The strategies adopting covalently labeled aptamers are commonly classified as: assays based on target binding-induced conformational change of the aptamers, assays based on target binding-induced aptamer-complementary strand displacement, and sandwich assays [50,90]. For the label-free approaches, the target–aptamer interaction is detected using redox molecules that interact diffusively or associate electrostatically with the surface-bound aptamers [90,106]. In addition, several electrochemical stripping methods have also been reported [108,109].

#### 3.2.1. Strategies Based on Target Binding-Induced Aptamer Conformation Change

**Figure 1:**
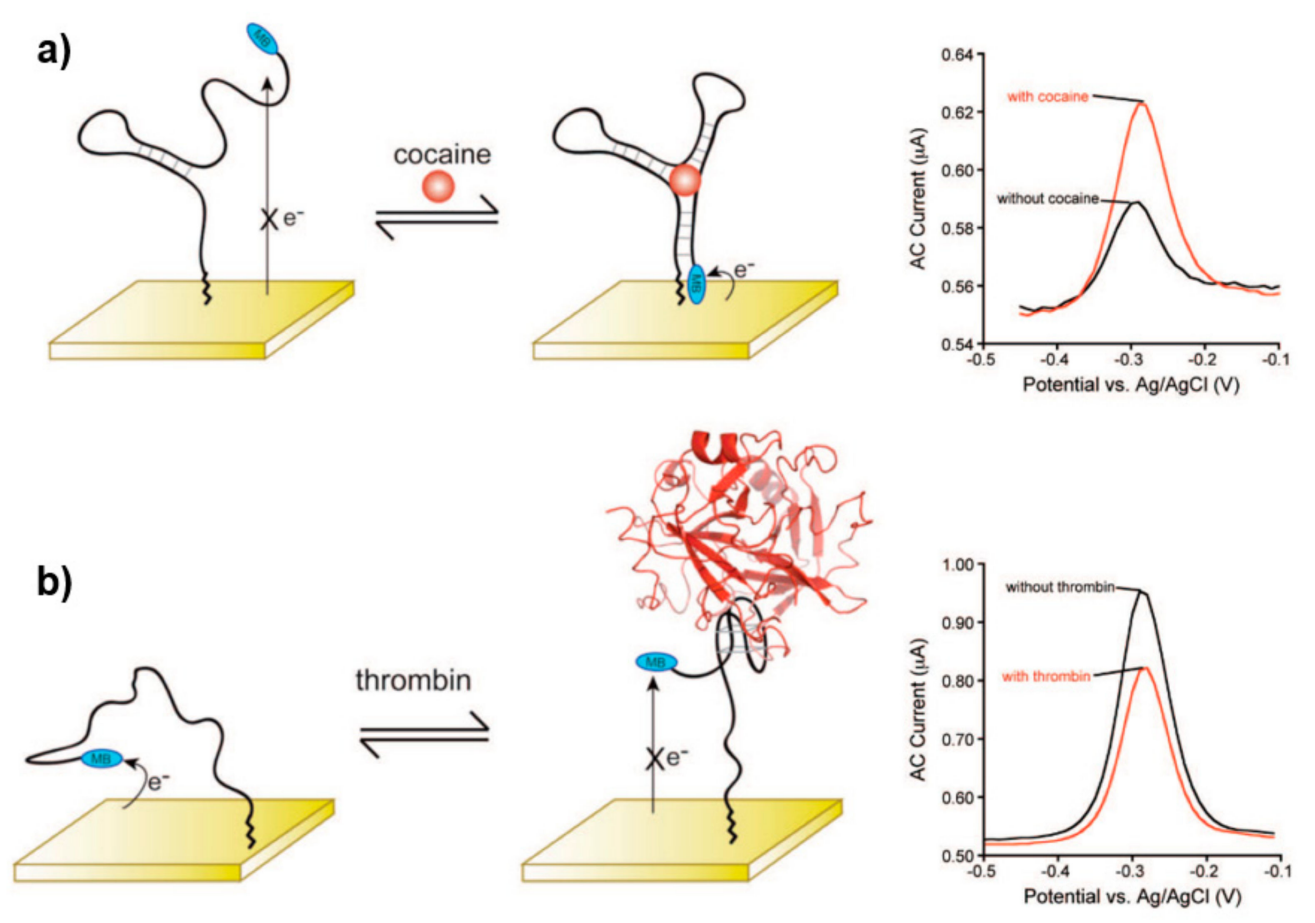
Schematic representation of (**a**) the “signal-on” strategy involving a methylene blue (MB)-tagged cocaine aptamer immobilized on a gold surface: the addition of the target induces the folding of the aptamer, bringing the redox tag near the electrode and increasing response current; (**b**) the “signal-off” strategy involving a MB-tagged thrombin aptamer immobilized on a gold surface: upon target binding, the aptamer undergoes a conformational change resulting in a decrease of the redox current. Reprinted with permission from [94]. Copyright 2008 American Chemical Society.

The two situations encountered in this case are the “signal-on” strategy, where the interaction of a redox-labeled aptamer with its target generates a change in aptamer conformation favoring the approach of the redox tag to the electrode surface, and the reverse case, the “signal-off” strategy, where upon target binding the distance between the redox label and the electrode increases due to the conformational change [90,94]. Two representative examples of electrochemical aptasensors depicting these situations have been presented in a study by Plaxco’s group [94]. In the first case, illustrated by an aptasensor for the small molecule cocaine (Figure 1a), the electron transfer is facilitated by the target’s presence and thus a monotonous increase in response current with the target concentration is obtained. Inversely, for the “signal-off” case, provided here for the thrombin protein aptasensor (Figure 1b), the electron transfer process is suppressed, so the response current is inversely proportional to the target concentration. In fact, one of the first studies illustrating this electrochemical strategy was produced by the same research group who constructed an aptasensor for thrombin, and it was based on a similar principle as that used with the optical aptamer-beacon sensors previously reported [110].

#### 3.2.2. Strategies Based on Target Binding-Induced Aptamer-Complementary Sequence Dissociation

**Figure 2:**
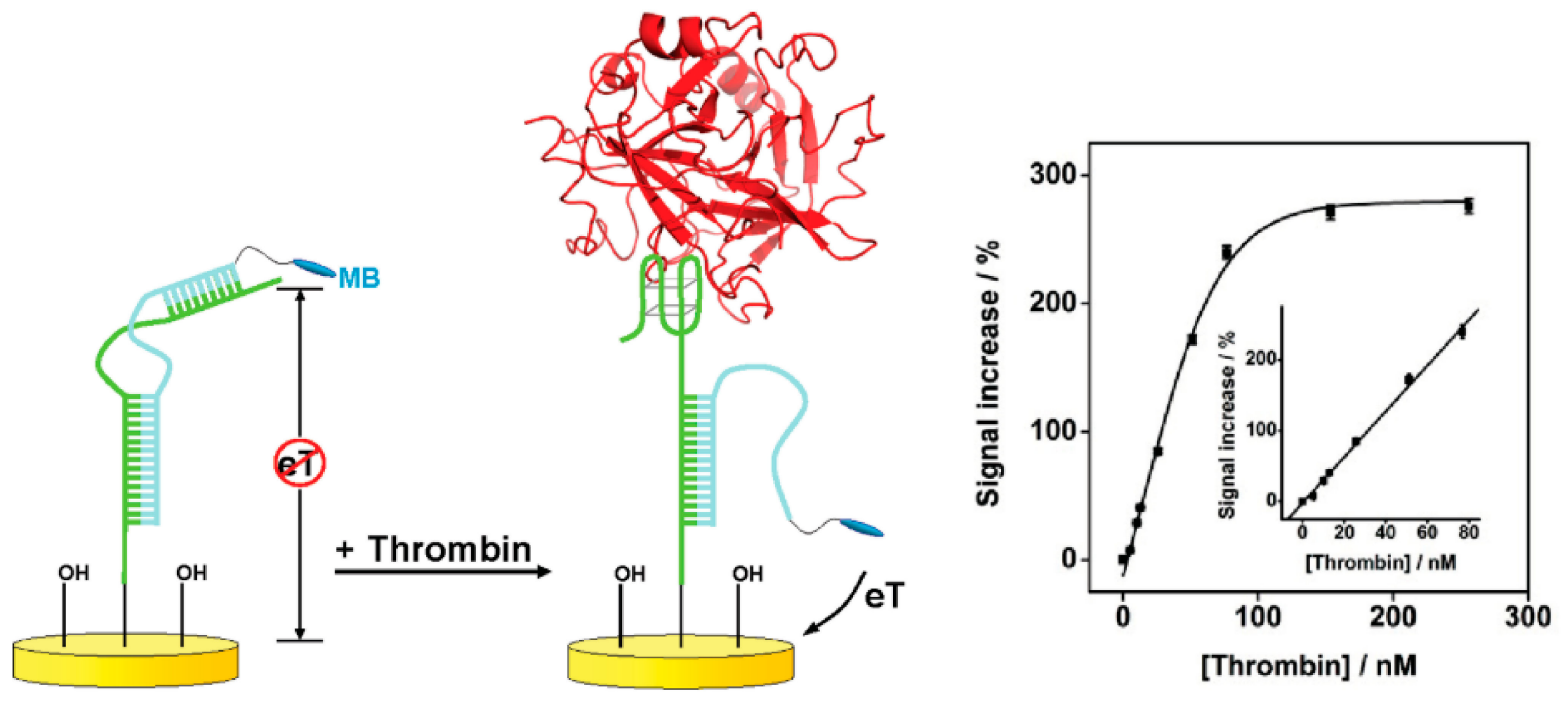
The schematic representation of the “signal-on” strategy involving a MB-tagged cDNA thrombin aptamer immobilized on a gold surface: upon target binding the aptamer undergoes conformational folding that loosens the tagged end of the cDNA, which approaches the electrode surface and generates a detectable redox current. Reprinted with permission from [112]. Copyright 2010 American Chemical Society.

These design strategies employ an antisense oligonucleotide (cDNA) complementary to the aptamer, and the analytical signal can be generated through both “signal-off” and “signal-on” mechanisms. In the first case (the “signal-off” strategy), the aptamer immobilized onto the electrode forms a dsDNA structure with a redox-tagged cDNA strand. In this structure, the redox tag is located in the electrode’s proximity, generating an electrochemical signal. When the target is added, the cDNA sequence is released and the redox signal decreases. In the “signal-on” strategy, a redox-tagged cDNA sequence is immobilized onto the electrode and forms a dsDNA complex with the aptamer, in which the redox tag is positioned away from the electrode. When the target is added, the aptamer strand is released, and the redox tag approaches the electrode surface, thus increasing the redox signal. Several variants of these designs have also been reported. For example, one of the first reports on this “signal-on” strategy employed a partially complementary oligonucleotide that formed a dsDNA complex with the electrode-immobilized aptamer [111]. As the aptamer folds when the target analyte is added, the tagged end of the cDNA becomes loose and, by approaching the electrode surface, it generates a detectable redox signal (Figure 2) [112].

#### 3.2.3. Sandwich Assays

**Figure 3:**
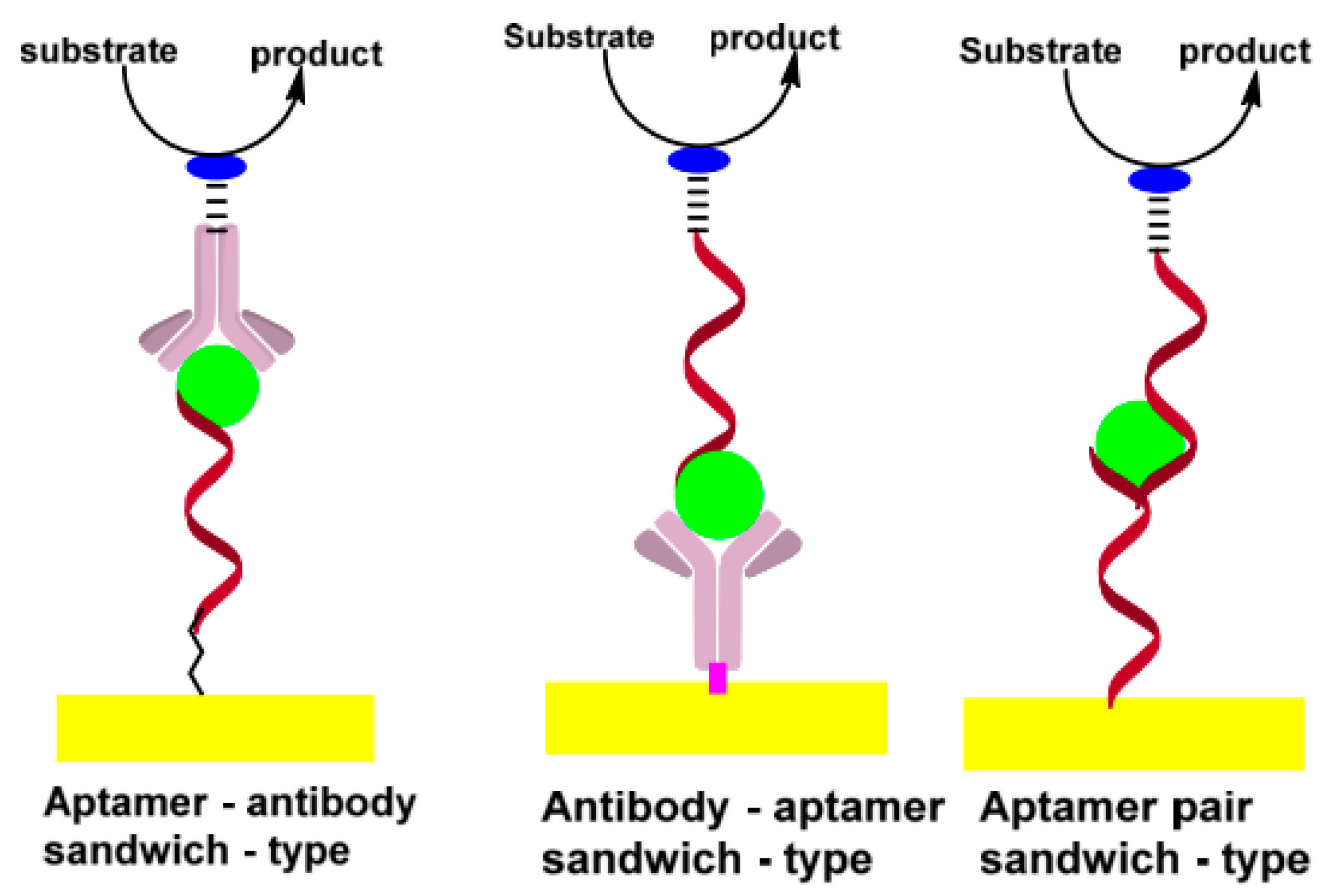
Schematic representation of sandwich-type structures employed in aptasensing assays. Reprinted from [114].

This strategy uses two bioreceptors for detecting the target, one immobilized onto the electrode surface (the capture element), and the other labelled with enzymes or nanomaterials in order to function as signaling element (reporter probe). This approach may involve antibody–aptamer sandwich sensing layers or a pair of aptamers (Figure 3). The prerequisite for obtaining a sandwich assay is that the analyte carries two epitopes, so that both receptors can bind simultaneously without affecting the two separate binding events [113]. The sandwich format commonly provides higher selectivity than single-receptor assays and can be assisted by a large variety of amplification methods [114].

#### 3.2.4. Strategies Based on Label-Free Approaches

**Figure 4:**
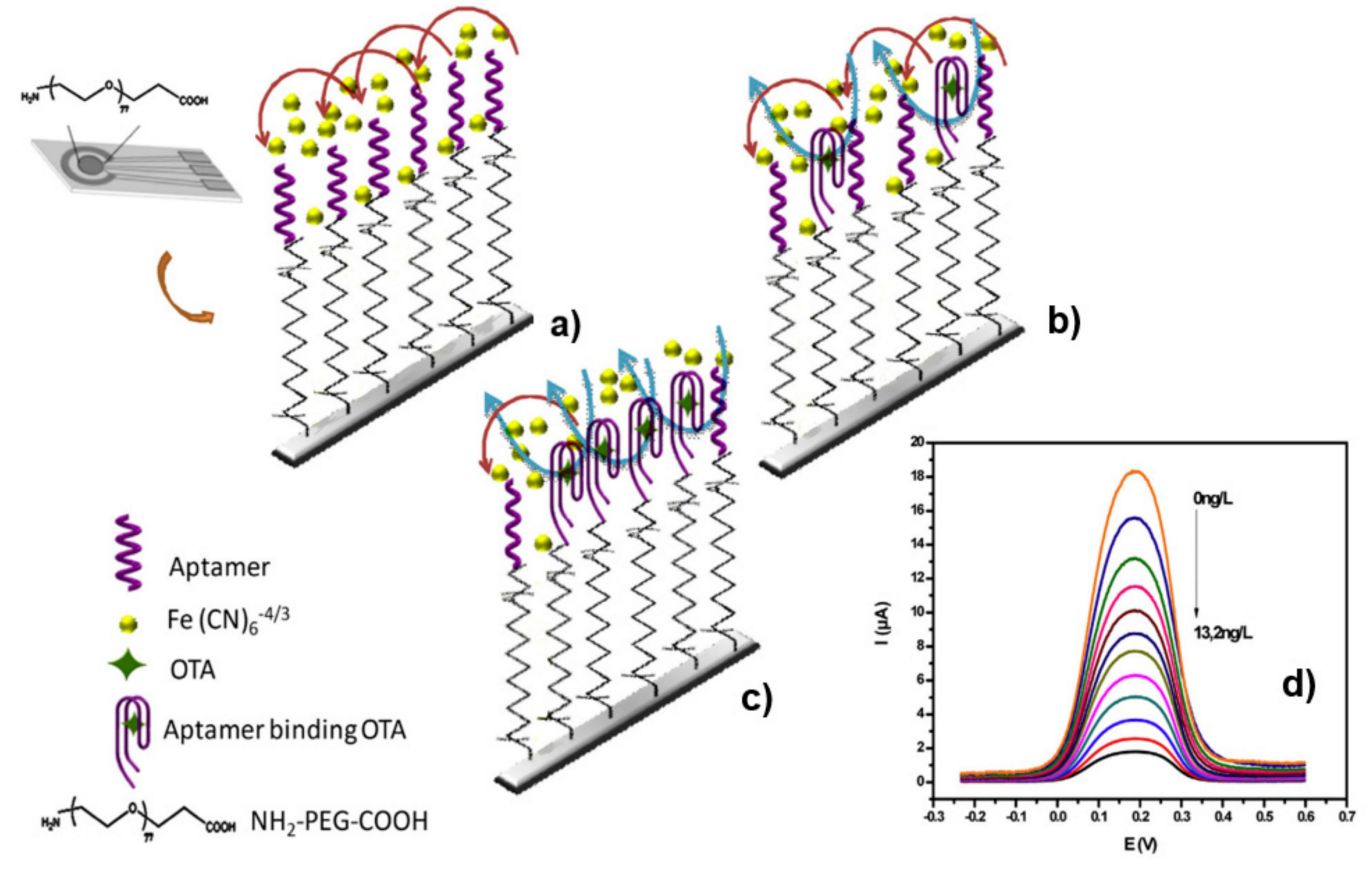
Schematic representation of a strategy involving unlabeled aptamers for the fabrication of an electrochemical aptasensor for ochratoxin A: (**a**) efficient electron transfer between the redox probe and electrode in the absence of the analyte; (**b**) inhibition of the electron transfer induced by the aptamer conformational change upon analyte binding; (**c**) further blocking of the electron transfer upon the additional increase in analyte concentration; (**d**) square wave voltammograms (SWV) depicting the decrease of the redox current with the analyte concentration. Reprinted with permission from [115]. Copyright 2015 Elsevier.

These strategies employ redox species that can diffusively interact or intercalate with the immobilized aptamers due to electrostatic interactions. Such a detection scheme was employed in the design of a label-free electrochemical aptasensor for ochratoxin A (Figure 4) [115]. As depicted in Figure 4a, in the absence of the target analyte the aptamer strand is usually unfolded and, therefore, the electron transfer between the [Fe(CN)_6_]^4−/3−^ redox probe and electrode is efficient. Upon analyte binding, the aptamer undergoes a conformational folding (G-quadruplex structure) which induces an inhibition of the electron transfer both by steric hindrance, and electrostatic repulsion between the negatively charged backbone and the redox probe (Figure 4b). An additional increase in the concentration of the analyte resulted in further blocking of the electron transfer (Figure 4c) and thus a further decrease in the electrochemical signal (Figure 4d). For EIS-based sensors, an increase in charge transfer resistance (R_ct_) upon target binding is usually observed, while for amperometric sensors, a decrease in the redox current is registered.

Other label-free approaches exploit the electrostatic interaction between redox cationic complexes like [Ru(NH_3_)_6_]^3+^ (RuHex) and the negatively charged phosphate backbone of the oligonucleotides. Typically, these methods are employed for assessing the surface density of DNA strands through chronocoulometry, as first reported by Steel et al. [116], by assuming complete charge compensation between the DNA phosphate residues and RuHex cations [66,88,116,117]. Furthermore, this approach permits the direct evaluation of the analyte concentration through monitoring the electrochemical response of surface-bound RuHex, as analyte binding is usually accompanied by a decrease in the integrated charge [118].

#### 3.2.5. The Ratiometric Electrochemical Strategy

**Figure 5:**
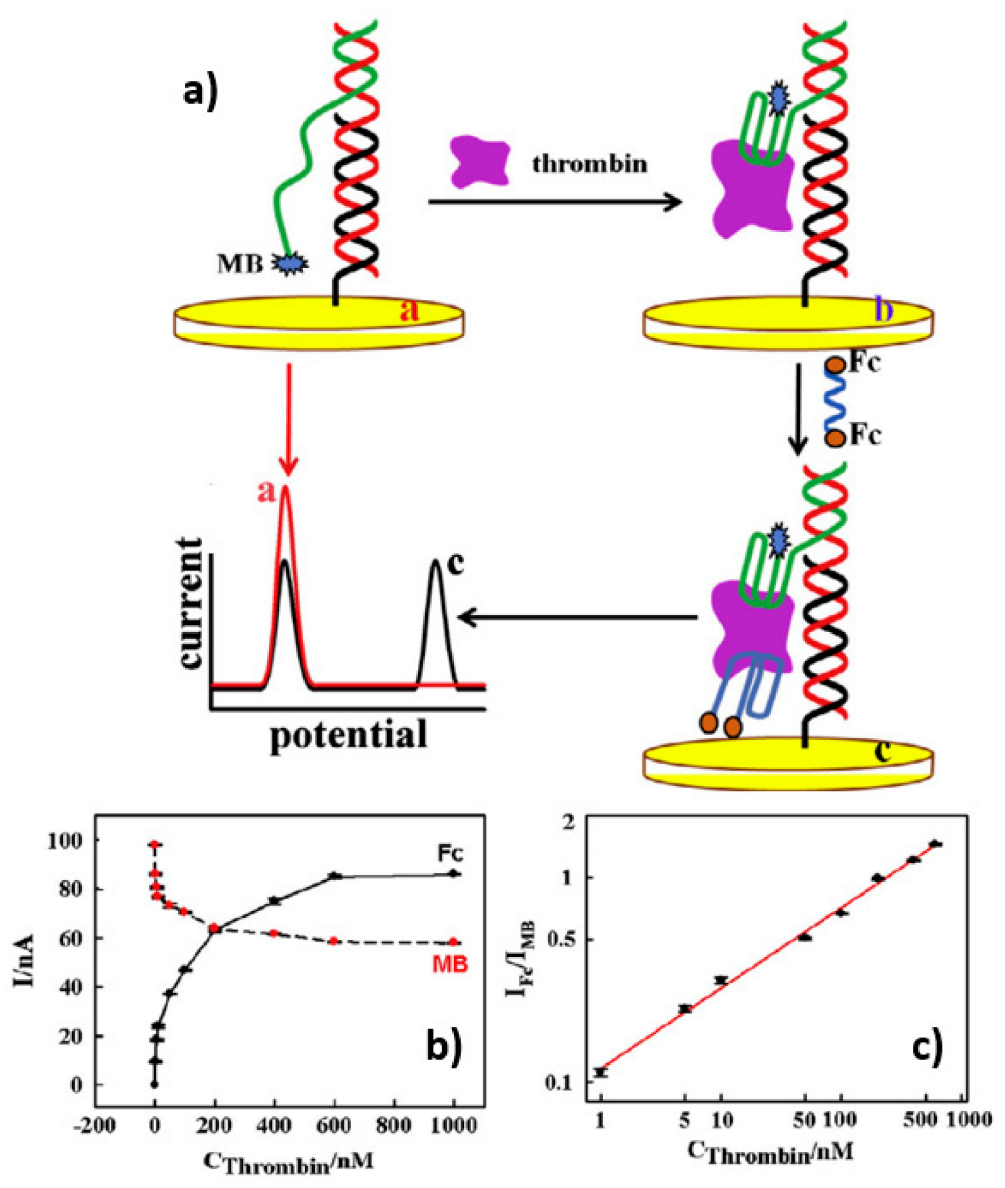
(**a**) The schematic illustration of the ratiometric electrochemical aptasensor for thrombin detection; (**b**) the relationship between the SWV peak current of MB and Fc and the thrombin concentration (linear scale); (**c**) the dependence of the peak current ratio I_Fc_/I_MB_ on thrombin concentration (logarithmic scale). Reprinted with permission from [123]. Copyright 2014 Elsevier.

These electrochemical sensors are based on the ratiometric approach, a widely used detection mode in fluorescence analysis [119], providing superior reliability, accuracy, and reproducibility [120,121]. Ratiometric electrochemical sensors display two response signals, and the analyte is quantified by the ratio of these two signals [121]. As emphasized in recent reviews, the difference between a dual-signal electrochemical sensor and a ratiometric sensor is that the dual-signal sensor’s output is in fact the sum of the two electrochemical signals and not their ratio [120,122]. One of the first ratiometric electrochemical aptasensors was reported by Yu et al. in 2014 [123], and their design was based on a sandwich structure consisting of a pair of thrombin aptamers, one labeled with MB and the other with ferrocene (Fc) redox tags (Figure 5). As a result of conformational changes brought by target binding, the aptamer labeled with MB functioned as a “signal-off” element, while the other functioned as a “signal-on” element (Figure 5a). Accordingly, the Fc signal increased and the MB signal decreased (Figure 5b), giving a linear dependance between the logarithms of the current ratio and thrombin concentration (Figure 5c) [123].

However, according to the latest reports, increasing the number of redox-active labels, as in the case of a ratiometric procedure, leads only to a minimal boost in sensitivity [120,121]. A significant increase in sensitivity can be achieved through amplification strategies, which are compatible with ratiometric electrochemical detection methods [120].

#### 3.2.6. Other Amplification Strategies Employed in Electrochemical Aptasensors

Since in real food samples many contaminants occur in trace amounts, adding a signal amplification feature in the aptasensor design allows their accurate detection even in the presence of excess interfering species. Traditional amplification strategies include labeling amplification, where a single nanomaterial-based tag incorporates numerous signal-generation elements, and target amplification, where a single target generates many signaling molecules through a multitude of analyte-recognition events [120]. Numerous aptasensing studies focused on food contaminants have employed the first strategy, based on nanomaterials, such as carbon nanomaterials (CNs) [28,96,124,125] and metal nanoparticles [125,126,127], or enzyme labeling [69,79]. The second signal amplification strategy aims to increase the signal/target concentration ratio, as in the case of nuclease-based target recycling [127]. Basically, after binding to the target, the aptamer is digested with nucleases, triggering the release of the target in order to initiate a signal amplification cascade [127,128]. Recently, more sophisticated DNA structures have been explored for signal amplification, like DNA walkers, DNA robots, and DNA tweezers [129].

## 4. The Role of Diazonium Electrochemistry for Aptasensors Development

### 4.1. Basic Considerations and Challenges Regarding the Aryldiazonium Grafting Strategy

The mechanism of electrografting with aryldiazonium salts has been extensively investigated since its first demonstration by Pinson [130] for the modification of carbon surfaces: it involves a reductive electron transfer to the aryldiazonium cation and the concerted cleavage of dinitrogen, which leads to highly reactive aryl radicals at the electrode vicinity followed by the formation of covalent bonds with the surface [131,132]. The covalent grafting of aryl groups is possible because the aryl radicals are not reduced at the potential they are generated, so they can react with the surface [130]. The usefulness of this strategy in biosensor development was recognized early on, in a pioneer study describing the covalent immobilization of glucose oxidase at glassy carbon (GC) electrodes functionalized through electrochemical reduction of the aryldiazonium salts [133]. The versatility of this modification procedure derives from the multitude of experimental alternatives for conducting the process, such as the substrates that can be modified or the variety of functional groups on the aromatic ring [31,134,135,136]. One of the most frequently reported aryldiazonium salts to be grafted is 4-nitrophenyldiazonium tetrafluoroborate. This commercially available derivative became a model for defining the grafting conditions and for evidencing new concepts in aryl diazonium electrografting studies. Its extensive use can be attributed to the -NO_2_ group, which has characteristic spectral (IR, XPS) [137] and electrochemical signatures and can be easily converted to -NH_2_, allowing further coupling of various bioreceptors [138]. Generally, the immobilization of various target molecules requires that the aryldiazonium salt possesses reactive functional groups [139]. For example, a great number of studies focused on biosensing applications employ substrate electrodes grafted with carboxyphenyl groups, which allow the covalent attachment of biomolecules through amide bonds, reactions mediated by carbodiimides such as 1-ethyl-3-(3-dimethylaminopropyl)-carbodiimide (EDC) [140]. Although typical grafting protocols employ diazonium salts synthesized in advance (usually tetrafluoroborates, generally stable at room temperature in their solid state) [141,142], the in situ generation of aryl diazonium salts is also possible. In this case, the diazonium compound is generated in the grafting solution through diazotization of the corresponding aromatic amine, either in aqueous acid with NaNO_2_ [143] or in aprotic solvents with reagents such as nitrosonium tetrafluoroborate [144], *tert*-butyl nitrite [145], or isoamyl nitrite [146,147,148]. Generally, the grafting process is conducted in acetonitrile or aqueous acid solutions, with minimal differences between the grafting in the different solvents [149]. The derivatization can be performed through electrochemical reduction, with chemical reducing agents [150,151,152], and can even occur spontaneously [153,154,155,156,157,158,159,160]. Spontaneous grafting is related to the reducing properties of the substrate and occurs on various metals, such as iron and copper, but also on carbon-based substrates [153,154,155,156,157,158,159,160]. However, several studies revealed that the substituent in the *para*- position has a strong influence on the proneness for spontaneous grafting, and salts with electron-withdrawing substituents are able to spontaneously graft even onto less-reducing substrates, such as carbon [132,161]. Among the different functionalization methods, the electrochemical reduction of aryldiazonium salts is the most popular since it allows a higher degree of derivatization control, and the process is straightforward and expeditious [36,131,161,162,163,164,165]. The potential of the characteristic diazonium electroreduction peak evidenced in cyclic voltammograms (CVs) is useful for assessing diazonium reactivity, as it is directly influenced by the nature of substituents on the aromatic ring [132,141,161,163,164,165,166,167]. Thus, the reduction potentials of salts substituted with electron-donating *para*-substituents are highly shifted toward cathodic values, while moderately or strong electron withdrawing groups, like -COOH or -NO_2_, shift the reduction potential towards more positive values [161,163,168]. Usually, the grafting process is conducted with CVs or potentiostatic methods, at potentials equal or more cathodic than the onset potential for diazonium salt reduction [31,161].

From the perspective of applications related to electrochemical biosensors, electrochemically assisted covalent grafting is preferred since deposition conditions can be easily controlled and thus significantly improve the overall reproducibility of the fabrication process [34]. Still, as commonly acknowledged, the major drawback of electrografting procedures is the high reactivity of electrogenerated aryl radicals, which react unselectively with the substrate and surface-grafted aryl groups. This usually leads to polyaryl layers with disordered structure and uncontrolled thickness, which are less suitable for building adequate sensing platforms [169]. The approaches reported so far to limit the formation of polyaryl layers have been reviewed elsewhere [35] and therefore only a brief inventory will be given here. Among these strategies, the most promising rely on diazonium salts bearing bulky substituents or protecting groups which can be cleaved after performing the grafting procedure. For example, the “formation–degradation” approach employs diaryl disulfide diazonium derivatives to obtain thiophenolate monolayers at various carbon surfaces, through electrochemical reduction followed by reductive cleavage of the disulfide bonds [170,171]. Similarly, the “protection–deprotection” approach employs diazonium salts with ethynyl substituents protected by trialkylsilyl groups, in order to obtain ethynyl-functionalized aryl monolayers after the deprotection step [172,173,174]. The latter method is able to promote a controlled spatial immobilization of various bioreceptors through the Cu(I)-catalyzed 1,3-dipolar cycloadition (“click”) reaction. Another popular procedure to suppress the formation of polyaryl layers uses radical scavengers during the electrografting step to capture the excess aryl radicals [175,176]. Although early studies employing 2,2-diphenyl-1-picrylhydrazyl (DPPH) as radical scavenger suggested that DPPH couples with aryl radicals generated from diazonium reduction, a recent study established that DPPH acts more like a redox mediator than a radical trapping species [169].

Another common limitation caused by the high reactivity and lack of selectivity of electrogenerated aryl radicals is the difficulty of controlling the surface composition of layers obtained through electrografting from mixtures of diazonium salts [177]. Tuning the relative ratio of grafted species can be achieved by adjusting their concentration in the grafting solution [177,178,179], or by applying two-step strategies consisting of successive electrografting of different derivatives. Apparently, the main constraint of the route based on successive electrografting is the difficulty of retaining both the electrochemical activity and the functional properties of the layer grafted first [177]. Leroux et al. obtained such binary films on GC electrodes by first grafting a phenylethynyl layer with triisopropylsilyl protecting groups which, after deprotection, led to nanometric pinholes [177]. These pinholes were electrochemically accessible for grafting with a second aryldiazonium derivative, while the coupling of the main functionalities could be achieved with a “click” reaction at the deprotected alkyne groups. Such an approach for electrode surface modification with binary aryl layers was applied by Marty’s group in the construction of a highly sensitive label-free ochratoxin impedimetric aptasensor [40].

### 4.2. The Role of Aryldiazonium Salts in Aptamer Immobilization on the Sensing Interface

As mentioned above, the aryl layers grafted by electrochemical reduction of diazonium salts allow the immobilization of ordered oligonucleotide probes onto the electrode substrate, thus increasing the efficiency of the recognition process [138]. Despite the fact that controlling the surface composition of mixed layers is challenging, diazonium electrografting offers advantages in terms of its simplicity, short preparation time, and long-term stability when compared with methods that rely on the self-assembly of alkanethiols [88]. Furthermore, aryldiazonium grafting can be performed on a broad range of electrode materials, hence providing wider opportunities for fabrication of electrochemical biosensors [180]. While carbon-based electrodes (GC, pyrolytic graphite, screen-printed carbon electrodes, carbon nanomaterials, and diamond) are undoubtedly the most suitable substrates for aryldiazonium grafting due to the formation of the stable C-C bonds, this approach has also been successfully applied for silicon [181], metals [182], and indium tin oxide (ITO) surfaces [183]. As can be seen in Table 2, which presents an analytical summary of electrochemical aptasensors developed through diazonium chemistry for the detection of food contaminants, the most frequently encountered functional group in biosensing applications is carboxyphenyl, as it allows the immobilization of biorecognition elements bearing -NH_2_ groups by means of carbodiimide-mediated coupling. Another group suitable for immobilization of biomolecules is aminophenyl, which can be either grafted directly or obtained through the reduction of nitrophenyl groups [36]. In fact, the first example of aryldiazonium functionalization employed for the preparation of nucleic acid-based biosensors involved the attachment of a DNA probe onto screen-printed carbon electrodes (SPCEs) grafted with aminophenyl moieties that were further converted to phenyldiazonium functions [34,184]. The main benefit of this approach is that a phenyldiazonium-functionalized layer allows the immobilization of unmodified oligonucleotides, and this strategy has been successfully tested with several sensing platforms [184,185].

In addition to carboxyphenyl or aminophenyl moieties, aryldiazonium salts containing alkynyl [84] or maleimide [186] groups have also been grafted onto various substrates in order to obtain biodetection platforms. For instance, a very sensitive amperometric biosensor for lysozyme detection was fabricated by grafting a phenylethynyl layer on a reduced graphene oxide (RGO)/polyethyleneimine (PEI) composite electrode, followed by coupling the azide-functionalized lysozyme aptamer through the well-known Cu(I)-catalyzed “click” reaction [84]. Bedioui’s group also reported the “click” covalent anchoring of alkyne-functionalized aptamers at azidophenyl-grafted areas of a fluorinated thermoplastic material, patterned selectively through SECM-assisted carbonization [187]. The immobilization of short recognition ssDNA sequences in phenylmaleimide groups grafted onto carbon nano-onions (CNOs) electrodes through thiol–maleimide reactions has also been reported [186].

The sensitivity of electrochemical affinity biosensors is critically influenced by the surface packing of biorecognition molecules [93]. Although an increased aptamer surface density can be beneficial in some cases because it maximizes the number of available binding sites [14], some studies have revealed a sharp maximum in terms of target binding efficiency at intermediate densities [19,93]. For aptasensors using hairpin molecules as recognition entities, more dispersed packing provides more space for unfolding, leading in turn to more sensitive detection. Conversely, when aptamers are packed too closely, they are unable to unfold properly due to steric hindrance [188]. In this respect, the use of diazonium-grafted functionalized aryl groups as anchors for aptamers proved very efficient in controlling the surface density, orientation, and stability of biorecognition molecules [34,36]. In particular, electrografting using protected aryldiazonium salts has demonstrated its effectiveness for the preparation of controlled structure monolayers [172,174,177]. For example, for a series of protected phenylacetylenes, changing the size of the protecting group from trimethylsilyl to triethylsilyl and tri(isopropyl)silyl ensured the formation of functional monolayers and allowed the fine-tuning of the surface coverage [172]. As discussed in Section 3.2.4, the aptamer surface density is typically measured by chronocoulometry in solutions containing [Ru(NH_3_)_6_]^3+^ [66,88,116,117]. A recent study using an innovative imaging method that combined RuHex binding with surface plasmon resonance (SPR) demonstrated that optimal surface densities for target binding depend on the size of the target molecule [93].

### 4.3. The Role of Aryldiazonium Chemistry in Multiplexed Detection

**Figure 6:**
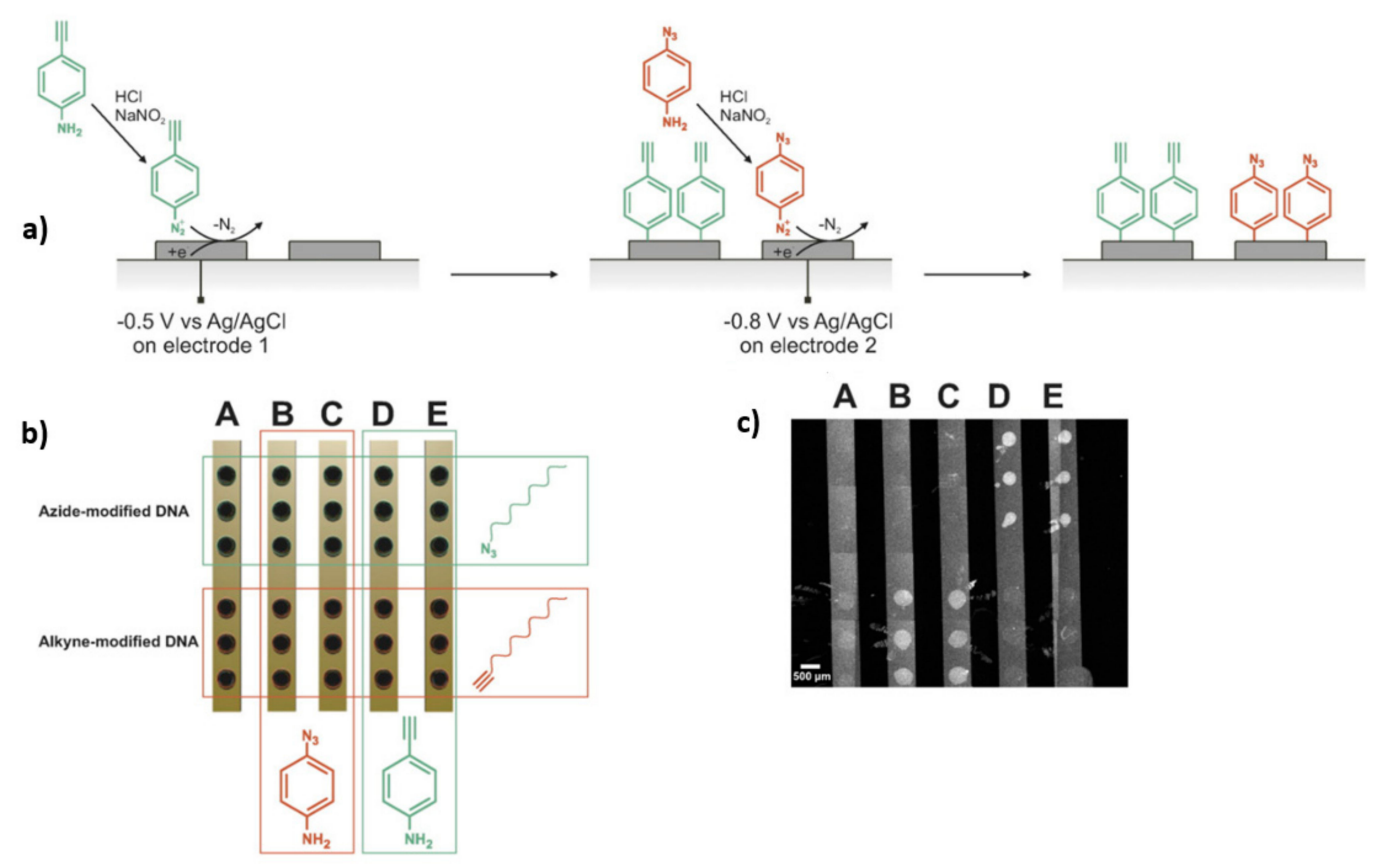
Schematic representation of the strategy employed for site-specific immobilization of two different ssDNA probes on a single chip: (**a**) site-specific electrografting of ethynylphenyl and azidophenyl groups on adjacent Au electrodes; (**b**) pattern for selective immobilization of azide- and alkyne-modified ssDNA: electrode A was not grafted and served as a reference, electrodes B and C were grafted with azidophenyl and electrodes D and E with ethynylphenyl groups, respectively; (**c**) fluorescence microscopy images taken after hybridization of immobilized ssDNA probes with Cy3 dye-labeled complementary DNA. Reprinted with permission from [38]. Copyright 2016 Elsevier.

The ongoing need for increasing food safety leads to a high demand for rapid, highly efficient, and cost-effective methods for the detection of multiple food contaminants. Methods for simultaneous detection can achieve the analysis of multiple targets in a single sample aliquot, thus reducing analysis time and saving reagents and costs [189]. Again, aryldiazonium chemistry offers promising prospects for multiplexed electrochemical biosensors [190,191], and the possibility of selectively patterning various biomolecules, including ssDNA, on arrays consisting of individually addressable diazonium-modified electrodes was reported as early as 2007 [192,193]. Brozik et al. selectively functionalized part of the electrodes forming an array by grafting carboxyphenyl groups through diazonium reduction and then attached amino-functionalized ssDNA aptamers via carbodiimide coupling. The remaining electrodes were functionalized with antibodies through the direct reduction of an aryldiazonium–antibody conjugate, in order to obtain a bifunctional sensing platform able to selectively detect DNA and proteins [193]. Later, Levrie et al. reported a versatile and simple method to immobilize different ssDNA probes on a multi-electrode chip (Figure 6) by exploiting the electroaddressability of diazonium electrografting and the chemoselectivity of “click” reactions [38]. Two different aryl groups (ethynylphenyl and azidophenyl) were grafted on adjacent electrodes, followed by the chemoselective coupling of alkyne- and azide-modified ssDNA oligonucleotides onto the functionalized surfaces. Due to the reduced background signal, such bifunctional interfaces proved their benefits for the detection of target analytes in complex matrices, such as food [35].

### 4.4. The Role of Aryldiazonium Chemistry in Increasing the Selectivity of Aptasensors

When aryl diazonium salts became extensively used for the modification of various substrates, it was realized that the barrier properties of electrochemically grafted aryl layers can be beneficial in electroanalysis and other areas of electrochemistry [162,194]. Besides the fact that an electrochemical grafting strategy is able to promote proper surface density, distribution, and stability for the aptamer recognition layer, it can also provide other key components; for example, an antifouling layer which ensures the enhanced selectivity of the analytical method. As reviewed recently by Jiang et al., diazonium salts bearing ethylene glycol (EG) or various zwitterionic groups (e.g., phosphoryl choline (PC), sulfobetaine (SB), or carboxybetaine (CB)) can be grafted on the sensing interface in order to confer antifouling properties [195]. It was reported that organic coatings consisting of mixed aryl layers bearing oppositely charged functional groups can form a hydrated layer by electrostatic interaction with water molecules, leading to a decrease in non-specific adsorption while allowing the Faradaic processes to proceed at the substrate electrode [35,196,197]. Therefore, this capability of zwitterionic moieties electrochemically grafted through diazonium chemistry can serve as an instrument to improve the selectivity of aptasensing devices and at the same time increase their stability. Such a strategy employing bifunctional layers was successfully applied in designing an amperometric platform for the detection of three genetic fragments of the New Delhi Metallo-β-Lactamase (NDM)-coding gene [182]. The sensor was fabricated using mixed aryl layers grafted onto the Au electrode surface by electrochemically reducing the diazonium salts of N-(4-aminobenzoyl)-N’-(4- maleimidobenzoyl)-1,2-ethylenediamine (AME) and 3-((4-aminophenyl)dimethylammonio)-propane-1-sulfonate (APSB). The AME layer promoted the attachment of thiolated ssDNA at the sensing interface, while the sulfobetaine-derived APSB layer ensured an efficient antifouling resistance for other DNA or enzyme complexes [182]. In another study published by Marty’s group, the immobilization of azido-functionalized aptamers onto an electrografted binary layer composed of nitrophenyl and ethynylphenyl groups allowed the fabrication of a highly sensitive and selective electrochemical aptasensor for ochratoxin A [40]. The increased sensitivity and the reduced non-specific signal were attributed to the binary layer obtained through aryldiazonium chemistry, and this strategy can be a simple way of preventing non-specific response.

### 4.5. The Role of Aryldiazonium Chemistry in Increasing the Stability of Aptasensors

As previously discussed, aryldiazonium chemistry emerged as an attractive alternative to the self-assembly approach, as it leads to more robust organic films than the analogous self-assembled monolayers formed through chemisorption of thiols on gold surfaces [198]. For example, Gooding’s group investigated the stability of grafted layers in terms of long-term storage, ability to withstand repeated cycling, and the potential window in which they remain on the surface, and found that layers obtained via electrochemical reduction of aryldiazonium salts are more tightly bound to gold than alkanethiol self-assembled layers [199]. The superior stability provided by aryldiazonium-grafted monolayers was also proved when stripping voltammetry sensors for heavy metal ions like Cu^2+^, Cd^2+^, and Pb^2+^ were fabricated by covalently attaching oligopeptides to surface-bound carboxyphenyl groups [199].

Similar results were reported by Civit et al., who analyzed the thermal stability of a series of mono- and dithiol-derived layers on Au surfaces in comparison with layers obtained through the electrochemical reduction of mono- and bi-functional diazonium salts. All the layers contained carboxyl groups and were further modified covalently with ssDNA probes, resulting in sandwich assays for the detection of human papillomavirus sequences. Their work demonstrated that sensing platforms fabricated using aryldiazonium-derived layers are highly suitable for temperature-regulated electrochemical DNA assays [200].

### 4.6. The Role of Aryldiazonium Chemistry in the Assembly of Nanomaterials at the Sensing Interface

The integration of nanomaterials in the design of biosensors offers additional benefits, such as increased electroactive surface area and high electron transfer rates, and at the same time affords the biocompatibility necessary for the successful immobilization of bioreceptors [19,201]. These features relate to the various functions that nanomaterials can serve in a biosensing device: transducer electrode materials, carriers for signaling elements, catalysts, or mediators that regulate the electron transfer processes [202]. Many detection schemes employ aryldiazonium-grafted nanomaterials as carrier tags or rely on substrate electrodes modified with nanomaterials through aryldiazonium chemistry for signal amplification [36]. For example, it has been demonstrated that diazonium electrografting leads to a very stable covalent anchoring of CNs on a wide range of conductive substrates [203,204,205]. Moreover, several studies have illustrated the enhancement of the electronic coupling between the electrode and sensing layer through the formation of stable graphene layers of controlled thickness on GC electrodes [205,206] or of robust carbon nanotube (CNT) assemblies on different substrates [203]. The covalent anchoring of CNs has been attained at various conducting surfaces modified with diazonium-generated layers containing carboxylic acid, amino, or hydroxyl functional groups [207]. Two procedures are typically adopted for anchoring CNs at carbon-based electrodes. The first consists of grafting the substrate electrode with nitrophenyl groups through diazonium reduction, followed by their electroreduction to aminophenyl, and the diazotization of the aminated surface in order to promote the covalent binding of CNs dispersed in solution [101]. Moreover, graphene oxide (GO) sheets can be anchored at aminophenyl-modified GC surfaces via π−π stacking interactions or electrostatic attraction between the negatively charged GO sheets and the positively charged aminophenyl groups [208]. Conversely, in the second approach, the CNs are grafted with nitrophenyl groups, which are reduced, diazotized, and finally grafted onto the substrate electrode. In a similar fashion, CNs can be functionalized with mercaptophenyl groups through the reduction of the corresponding diazonium salt, which facilitates their self-assembly on gold substrates via Au–S bonds. Such an approach was reported by Lian et al. for the fabrication of an aptamer/graphene interdigitated Au electrode for the piezoelectric detection of S. aureus in milk samples [209]. The development of sensing devices with high analytical performances could also benefit from both the intrinsic properties of CNs and their special arrangement, as in the case of vertical alignment of CNTs onto aryldiazonium-modified surfaces [210]. Rawson et al. demonstrated the vertical alignment of short, single-walled CNTs coupled to aryldiazonium-grafted ITO substrates, which were further modified with ssDNA and employed successfully for intracellular electrochemical sensing [211].

## 5. Electrochemical Aptasensors Developed Using Aryldiazonium Chemistry for Food Safety Monitoring Applications

To our knowledge, the electrochemical aptasensors developed using aryldiazonium chemistry for applications in food safety monitoring have not yet been systematically reviewed. In this section, we discuss a selection of promising electrochemical aptasensors for the most commonly encountered food contaminants that have demonstrated their applicability in real samples. An overview of their design, in terms of the aptamer immobilization strategy and detection principle, alongside their analytical performance (sensitivity, limit of detection, selectivity, and reusability) for each type of contaminant (including antibiotics, toxins, metal ions, pathogens, and other food contaminants) is also provided in this section and summarized in Table 2.

### 5.1. Electrochemical Aptasensors for Antibiotics

Antibiotics are antimicrobial drugs widely utilized for therapeutic purposes or as supplements in livestock feed. Their abuse can have serious consequences, such as the rapid emergence of resistant bacteria [22,212]. During the constant efforts to develop specific and sensitive analysis tools for the detection of antibiotics in contaminated food products and water, electrochemical aptasensors have received extensive consideration. A recent comprehensive review of aptamer-based biosensors for the different classes of antibiotics suggested the popularity of aptasensing assays based on electrochemical transduction for these analytes over other detection principles [213]. In this section, the particular case of electrochemical aptasensors prepared through diazonium chemistry is discussed. The application of the developed assay for real sample analysis is illustrated by the detection of tetracyclines, kanamycin, and penicillin G in milk samples at concentrations compatible with the legislation requirements.

#### 5.1.1. Tetracyclines

**Scheme 2:**
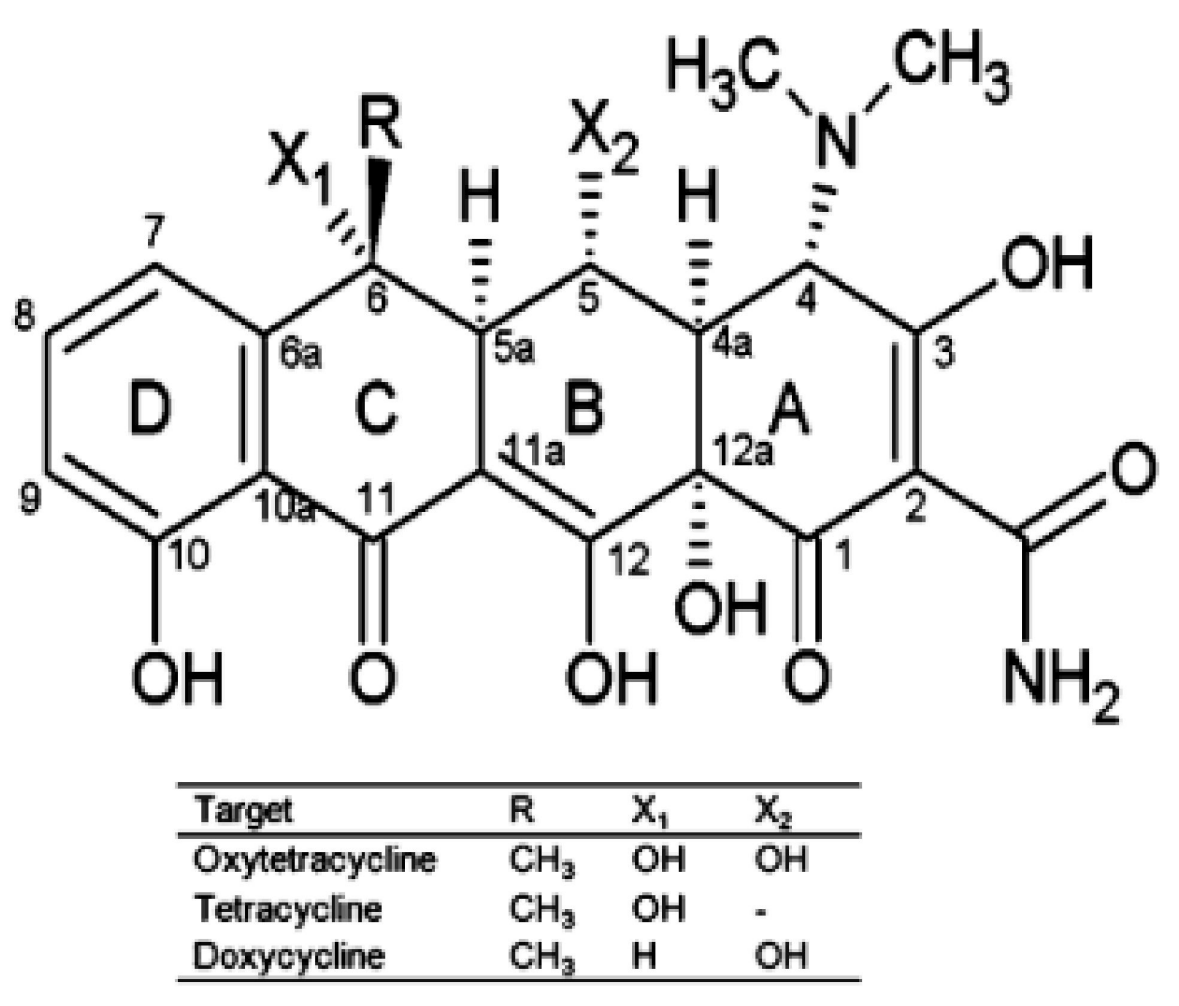
General structure of tetracyclines. Reprinted with permission from [60]. Copyright 2008 Elsevier.

The accumulation of tetracyclines (TCs), as well of other antibiotics, in dairy food products is the result of their extensive use in veterinary medicine and aquaculture for prophylactic and therapeutic purposes [214,215]. This poses a severe threat to human health, and the World Health Organization has established a maximum acceptable content of 100 μg kg^−1^ of tetracycline in milk and in animal tissues entering the human food chain [216,217]. The most commonly used TCs are oxytetracycline (OTC), doxycycline (DOX), and tetracycline (TET), for which substituents X_1_ and X_2_ are –H and/or –OH (Scheme 2) [60].

The majority of the developed aptasensors for tetracyclines are based on studies conducted by Gu’s group [60,218]. These employ common oligonucleotide sequences found in both tetracycline group-specific (TGS) and oxytetracycline-specific (OTC) aptamers, which might be required for binding to the common tetracycline ring system at carbon atoms 10, 11, 12, and 1 (Scheme 2) [60]. The group also developed aptamers that selectively bind to OTC and discriminate structurally similar compounds based on minor variations of functional groups such as –H or –OH at positions 5 and 6, as in tetracycline and doxycycline [62,218]. Such a 76-mer oligonucleotide aptamer for tetracycline [60] was employed by Alawad et al. for the fabrication of a label-free electrochemical aptasensor for reagentless determination of tetracycline (TET) in water [25]. The immobilization of the aptamers at the SPCEs’ transducer electrodes was achieved via carbodimide coupling at the carboxyphenyl moieties grafted by diazonium chemistry, and the detection of TET exploited the aptamer’s intrinsic electrochemical activity. The aptasensor displayed a linear domain ranging from 0.05 μg L^−1^ to 20 μg L^−1^, a detection limit (LOD) of 0.035 μg L^−1^, good specificity for TET, and could be successfully applied for the detection of TET in natural waters [25]. Recently, Yang et al. [61] discussed the design of a label-free electrochemical aptasensor for OTC with good stability, attributed to the covalent attachment of the aptamer on the sensing interface by diazonium chemistry. They adopted a signal-off strategy and quantified the analyte concentration by measuring, using differential pulse voltammetry (DPV), the oxidation signal of a redox couple present in the solution, which decreased as a consequence of analyte binding. The calibration curve obtained for a wide concentration range (1.0 μg L^−1^–1.0 × 10^5^ μg L^−1^) was presented in terms of the linear dependence of the DPV current on the logarithm of OTC concentration, while the LOD was 0.229 μg L^−1^. The sensor also displayed good selectivity and reproducibility, and its suitability for real milk sample analysis was demonstrated by spiking and recovery studies (mean recoveries: 87.0–99.3%, ±3.45%) [61].

Scanning electrochemical microscopy (SECM) has also been recently employed as a detection technique for electrochemical aptasensors [59]. In one published study, a specific TET aptamer [60] was immobilized by carbodiimide-mediated coupling onto a carboxyphenyl-modified SPCE by diazonium chemistry. The SECM imaging tool served to monitor the intrinsic redox activity of the aptamer and to evaluate the non-homogenous distribution of the aptamer on the electrode surface. This assessment was made by imaging the intense spots that appeared on the scanned area, consistent with the increase in the oxidation current of the aptamer recorded at the UME tip. Following TET binding, a substantial decrease in current was evidenced, a typical feature of the signal-off sensors [59].

#### 5.1.2. Kanamycin

Kanamycin is an aminoglycoside antibiotic with strong antibacterial activity against both Gram-negative and Gram-positive bacteria. There is evidence that consuming food containing residues of this drug may result in serious side effects, including loss of hearing, nephrotoxicity, and antibiotic resistance [63]. The maximum residue limit (MRL) of kanamycin in milk has been set at 150 μg kg^−1^ in the EU and 200 μg kg^−1^ in China [64,219]. Several electrochemical aptasensing assays have been reported in the last years for kanamycin detection in milk samples [24,63,219,220]. A disposable, label-free aptasensor was developed by Sharma et al. by employing diazonium chemistry for the aptamer immobilization. The authors emphasized the role of the electrochemically grafted carboxyphenyl moieties for coupling of the aptamer sequences in both improving the stability of the platform and providing a uniform surface modification that favored the assay sensitivity [63]. The binding interaction between the target and the aptamer was followed by the inhibition in the Faradaic response of a redox species in the solution, evidenced in EIS measurements by an increase of R_ct_. The sensor exhibited a dynamic range of 1.2–600 μg L^−1^, with linearity in the domain from 1.2–75 μg L^−1^ and an LOD of 0.11 μg L^−1^. It was also reported that this particular aptamer sequence has a high affinity for both kanamycin and its derivatives, such as kanamycin B and tobramycin [64]. Nevertheless, the aptasensor presented a good selectivity to kanamycin without interference from other aminoglycosides, like streptomycin and gentamicin, and the biosensor performance was successfully demonstrated by kanamycin detection in spiked milk samples (mean recoveries: 96.88–100.5%, ±4.56%) [63].

#### 5.1.3. Penicillin

Penicillin G (Pen-G), a β-lactam antibiotic and a member of the penicillin group of antibiotics, is extensively used as a therapeutic agent and veterinary drug [221]. The presence of penicillin residues in dairy products poses a serious health threat, which can lead to severe effects, such as drug allergies and generalized bacterial resistance. For these reasons, the European Union regulations established an MRL of 4 µg kg^−1^, the lowest MRL value among the β-lactams [222]. As in the case of other antibiotics, the development of accurate and fast detection methods for the quantitative monitoring of Pen-G residues in milk is crucial. As highlighted in a recent review, rather few aptasensors have been developed for the detection of Pen-G up to now [221]. The work performed by Paniel et al. described the selection of aptamers for penicillin using the Capture-SELEX technology for the first time, and they obtained a 78-base Pen-G-specific aptamer for the development of an impedimetric aptasensor [65]. The oligonucleotide capture molecules used as transducers were immobilized on screen-printed carbon electrodes (SPCEs) through diazonium chemistry. The developed device allowed the detection of penicillin at concentrations ranging from 0.4–1000 µg L^−1^ with an LOD of 0.17 µg L^−1^, which is compatible with MRL values, and with better performance in comparison with an ELISA method (concentration range: 4–400 µg L^−1^, LOD: 3 µg L^−1^). The sensor selectivity was tested by comparing its response to 4 µg L^−1^ Pen-G and other antibiotics (chloramphenicol, kanamycin, and tetracycline), including other two β-lactams (ampicillin and amoxicillin). The authors found that the aptamer could also bind the other β-lactams with a lower affinity, but not compounds belonging to other antibiotic classes. Moreover, the suitability of the developed sensor for real milk sample analysis was demonstrated by spiking milk samples with penicillin in a relevant concentration domain (mean recoveries: 83–100%, ±15%) [65].

### 5.2. Electrochemical Biosensors for Hormonal Disruptors

#### Estradiol

The growing concern about the effects of endocrine-disrupting chemicals has generated a demand for sensitive, reliable, and real-time detection methods in environmental and food samples [223]. These chemicals are able to enter the human body through ingested food and may cause adverse health effects even at low concentrations. For example, 17β-estradiol (E2), a natural estrogen, possesses the highest endocrine activity and causes damage to the endocrine system by disrupting the effects of natural hormones, thus triggering other immunological diseases, even at levels as low as 1 ng L^−1^ [96]. As E2 promotes animal growth rates and increases milk yield, it is abundantly used in the dairy industry [224].

The first aptamer for selective identification of E2, proposed in 2007 by Kim et al. [67], was a 76-mer ssDNA, while later studies investigated the performance of truncated versions of the original aptamer [66,96,224]. In a recent study, a diazonium grafting strategy was employed for the immobilization of a 38-mer amino-functionalized E2-aptamer on electrochemically reduced graphene oxide (ERGO)-modified electrodes. The use of ERGO as an amplifying platform combined with the synergic effect of the high affinity (K_d_ = 2.7 nM) and specificity of E2 aptamers enabled the femtosensitive electrochemical detection of E2 (LOD of 0.5 fM) in wastewaters and pharmaceutical dosage forms [66]. The detection principle was based on measuring the irreversible oxidation current of the hydroxyl group present in the aromatic ring of the E2 molecule using square wave voltammetry (SWV). The aptasensor displayed typical “signal-on” sensing features as, upon binding to the E2 target, the aptamers are folded into G-quadruplex configuration that forces the analyte into proximity with the aptasensing interface, increasing the electron transfer efficiency. The aptasensor displayed two domains of linearity for the oxidation current with the E2 concentration: 1.0 × 10^−15^–9.0 × 10^−12^ mol L^−1^ and 1.2 × 10^−11^–2.3 × 10^−10^ mol L^−1^. The good stability of the sensing interface was attributed both to the covalent attachment of the E2 aptamer by diazonium chemistry and the ERGO-based platform, which provided a biocompatible microenvironment for the biomolecules’ immobilization, stabilizing their activity [66]. The analytical results obtained after testing the sensor in E2-spiked wastewater and pharmaceutical dosage forms demonstrated its suitability for environmental and pharmaceutical sample analysis (recoveries for wastewater samples: 94.05–100.47%, ±2.2%; for pharmaceutical samples: 96.87–100.22%, ±3.3%).

### 5.3. Electrochemical Biosensors for Toxins

Mycotoxins are metabolites of fungi that may cause severe diseases and even death, and there is a continuous need for sensitive and selective sensors for these compounds [2]. The most well-known mycotoxins are produced by Aspergillus, Fusarium, Penicillium, and Alternaria fungi and belong to the classes of ochratoxins, aflatoxins (AFs), patulin (PATs), and fusarium toxins [225]. Mycotoxins can contaminate a wide variety of food products, like cereals, dairy products, grape juice, coffee, nuts, spices, cocoa beans, and wine, and because of their chemical and thermal stability, they cannot be simply destroyed during food processing operations [70,226]. Based on their carcinogenic risk, they are classified into different categories and, among them, aflatoxin B1 (AFB1) is a potent human carcinogen (group 1), while ochratoxin A (OTA) and fumonisin are possibly carcinogenic in humans (group 2B) [227]. Maximum permitted levels for the most prevalent and toxic mycotoxins in different types of food have been established worldwide [227].

#### 5.3.1. Ochratoxin A

**Figure 7:**
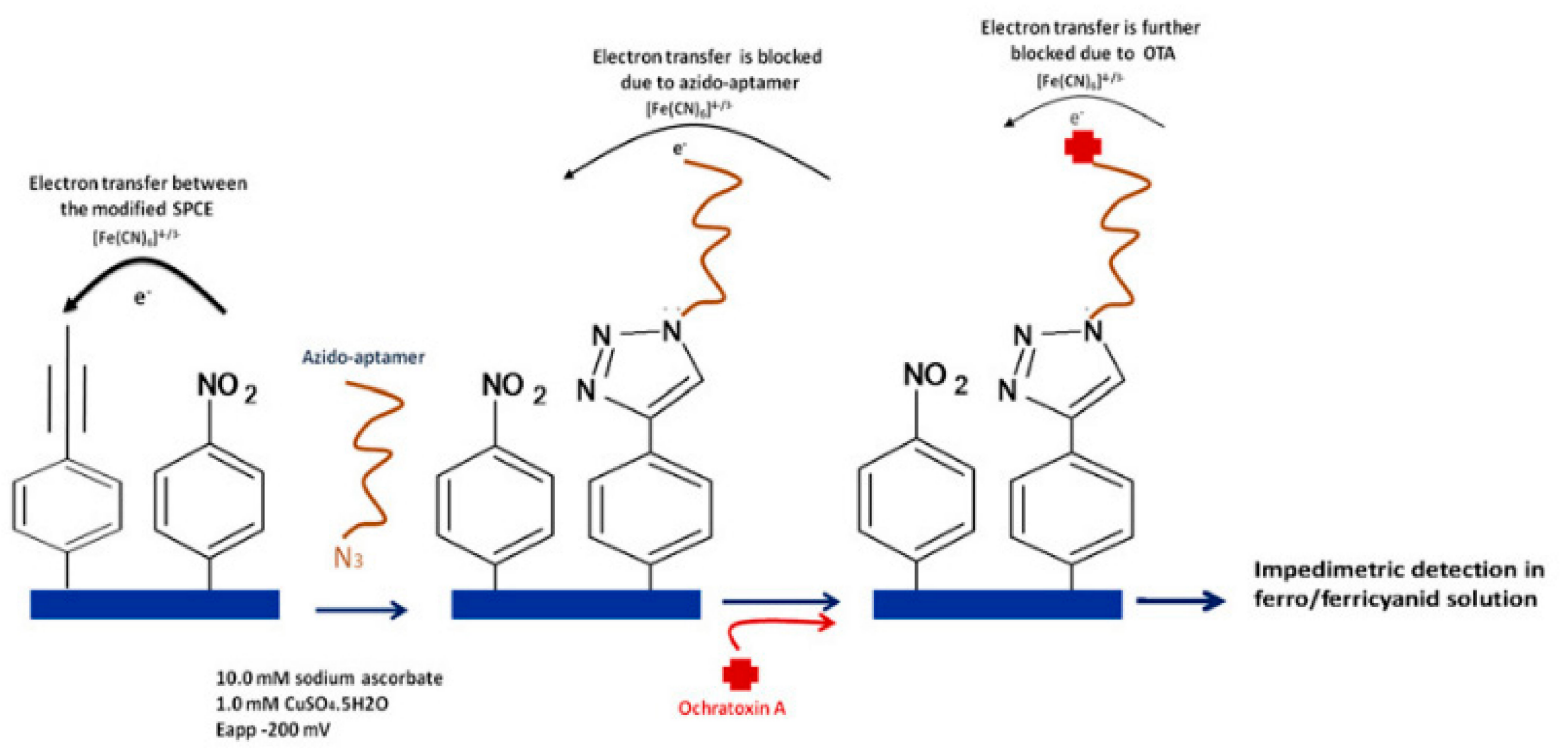
Design and working principle of an impedimetric aptasensor for OTA. The aptamer was immobilized through a “click” reaction at a phenylethynyl layer grafted through diazonium chemistry. Reprinted with permission from [40]. Copyright 2013 Elsevier.

Ochratoxin A is the most toxic member of the ochratoxin group and is encountered in a wide range of raw or processed food products, like cereals, grapes, coffee beans, cocoa-derived products, wine, and beer [68,228]. Depending on the type of food, the permissible levels of OTA established in the European Union are between 0.5 and 80 µg kg^−1^ [225]. In the last years, numerous approaches for the detection of mycotoxins, including ochratoxin, by utilizing electrochemical aptasensors have been reported [229]. The first aptasensing assay for the quantitative analysis of mycotoxins was presented by Cruz-Aguado and Penner, and the SELEX-obtained aptamers have been used for the determination of ppb quantities of OTA in naturally contaminated wheat samples [71]. In 2013, Marty’s group proposed different designs for OTA electrochemical aptasensors involving the immobilization of the bioreceptor probes onto organized mixed aryl layers grafted by diazonium chemistry at different substrates [40]. The first study employed a 36-mer aptamer [71] for the fabrication of a highly sensitive and regenerable electrochemical impedimetric aptasensor for the detection of OTA [40]. The SPCEs used as substrates were successively modified through electrografting of a protected phenylacetylene layer, followed by a second nitrophenyl layer. After deprotection, the acetylene groups were coupled with the azido-functionalized aptamer through “click” chemistry. The sensor response was measured by the increase in R_ct_ of Fe(CN)_6_^4−/3−^ species in the solution upon target binding (Figure 7). The sensor displayed linear behavior in the concentration range from 1.25 ng L^−1^ to 500 ng L^−1^, with an LOD of 0.25 ng L^−1^, and demonstrated good selectivity and reproducibility. As discussed in a previous section, the reduced non-specific adsorption was attributed to the presence of the binary aryl layer on the electrode surface. Moreover, the aptasensor was amenable to full regeneration. A mild regeneration solution [230] could be applied to renew the aptasensor up to at least 10 times, with no significant effect on the sensor response. The analytical reliability of the sensor for OTA detection in real samples was demonstrated by assays performed in OTA-spiked beer samples [40].

Numerous studies employing the same oligonucleotide immobilization technique have been reported since by the same research group [68,69,70,91]. A similar strategy, but employing a carboxyphenyl grafted layer for the aptamer immobilization through carbodiimide coupling, was used to obtain a label-free electrochemical impedimetric aptasensor for OTA in cocoa beans [68]. The aptasensor exhibited a linear response with an OTA concentration in the range of 0.15–2.5 μg L^−1^ and an LOD of 0.15 μg L^−1^. Moreover, OTA could be detected at concentrations as low as 2 μg kg^−1^ in cocoa samples, which meets the requirements imposed by EU regulations [68]. A more sensitive assay for detecting OTA in cocoa beans, based on a competitive aptasensor, has also been proposed [69]. In this approach, biotin-labeled and unlabeled OTA competed to bind with the aptamer immobilized at a SPCE electrode, and the detection was performed by adding avidin–alkaline phosphatase (ALP) enzyme complex and 1-naphthyl phosphate [69]. The ALP enzyme dephosphorylates the non-electroactive substrate 1-naphthyl phosphate to 1-naphthol, which is oxidized at the electrode surface and generates a signal measured by DPV. The selective detection of OTA, with a linear dependence of the response current with a concentration in the 0.15–5 μg L^−1^ domain and an LOD of 0.07 μg L^−1^, demonstrated the method’s suitability for the determination of this toxin in cocoa samples [69]. The relevance of the sensor for real sample analysis was proved by testing cocoa samples spiked with OTA (mean recoveries: 82.1–85.0%, ±5%).

Very recently, Marty’s group published a new study in which they adapted the previous method for fabricating the OTA impedimetric aptasensor using affordable pencil graphite electrode (PGE) substrates [70]. The well-known carbodiimide coupling has been used for binding amino-modified aptamers at the PGE surface, electrochemically grafted with carboxyphenyl layers. This simple-to-design aptasensor displayed a linear response in the 0.1–2 μg L^−1^ concentration range, with a very good LOD of 0.1 μg L^−1^, and was successfully tested in spiked beer samples, with OTA concentrations in the range from 0.4 to 1.6 μg L^−1^ (mean recovery of 93.4 ± 6.6%). The employment of diazonium electrografting for aptamer immobilization resulted in a high level of stability and reproducibility, thus recommending this facile yet powerful strategy for the design of low-cost aptasensors [70].

#### 5.3.2. Aflatoxin

Aflatoxins are another class of foodborne toxins that contain a coumarin ring and an unsaturated lactone moiety, and they are classified in several types, such as B1, B2, M1, M2, G1, G2, etc. [2,231]. They are mainly found in foodstuff contaminated with aflatoxin-producing Aspergillus or in dairy milk from animals that have consumed contaminated feed. The most prevalent are aflatoxin B1 (AFB1) and its metabolite aflatoxin M1 (AFM1), both considered highly carcinogenic and mutagenic [72,231]. In the European Union, the permitted concentrations of aflatoxin B1 (AFB1) are required to be in the 0.1–12 µg kg^−1^ range [3].

Following the same principles described for OTA aptasensors [68], disposable, cost-effective, and label-free aptasensors for AFB1 were developed by employing two aptamer sequences (a 35-mer sequence denoted seqA and 50-mer patented sequence denoted seqB [73]). The aptamers were immobilized by carbodiimide-mediated coupling on SPCE grafted with carboxyphenyl groups, and the transduction principle was based on the change in electron transfer resistance across the electrode–electrolyte interface upon target binding, measured by EIS. The analytical performance of the assays based on each aptamer was later compared in terms of LOD, linear range, and recovery values. The aptasensor based on seqB showed a higher sensitivity (0.271 (μg L^−1^)^−1^ vs. 0.193 (μg L^−1^)^−1^) and a wider linear range (0.1–16.0 μg L^−1^ vs. 0.25–16.0 μg L^−1^), while the aptasensor developed with seqA had a slightly better LOD (0.125 μg L^−1^ vs. 0.25 μg L^−1^). Both aptasensors were capable of detecting AFB1 with high sensitivity and reproducibility, and their applicability for real samples was demonstrated after AFB1 spiking of beer (mean recoveries: 96.0–102.0%, ±5.42%) and wine samples (mean recoveries: 92.0–101%, ±4.83%) [72].

AFM1 can be found in commercially available milk and is generally considered as one of the most serious problems for food safety [31]. Therefore, its concentration in milk is strictly regulated and the maximum permissible levels of AFM1 in milk and dairy products are set at 0.05 μg kg^−1^ in the European Union [232]. An impedimetric aptasensor for the determination of AFM1 in milk, employing the aptamer immobilization at a carboxyphenyl layer grafted by diazonium chemistry onto the surfaces of SPCEs, was developed by Istamboulié et al. [74]. Two previously obtained variants of the aptamer sequence were employed [75], functionalized with either an amino or amino-hexa(ethylene glycol) group at the 5’ end [74]. The use of the hexa(ethylene glycol) spacer allowed a more efficient immobilization of the aptamer, leading to an increase in assay sensitivity. The developed aptasensor responded to AFM1 in the concentration range from 0.02 to 1 μg L^−1^, which is compatible with natural contamination levels and in accordance with the limits fixed by European Union legislation. Real milk samples were successfully analyzed with the developed aptasensor, and the results correlated well with the ones obtained from a commercial immunoassay (98–114% recovery rates). It worth noting that these studies showed that pasteurization had no effect on the final concentration of AFM1 in milk [74].

#### 5.3.3. Patulin

Patulin (PAT) mycotoxin is a polyketide lactone, produced by various fungal species from the classes Penicillium, Aspergillus, and Byssochlamys [77]. Major potential sources of PAT are fruit- and vegetable-based products, especially apples [76]. PAT is considered the most important fruit toxin globally, and its acute and chronic toxic effects on animal and human health are well-known [77]. PAT levels in food are strictly regulated in the European Union, and the maximum permissible limits are 50 μg kg^−1^ in fruit juices and other beverages, 25 μg kg^−1^ in solid products, and even lower in labeled products for young children—10 μg kg^−1^ [233].

**Figure 8:**
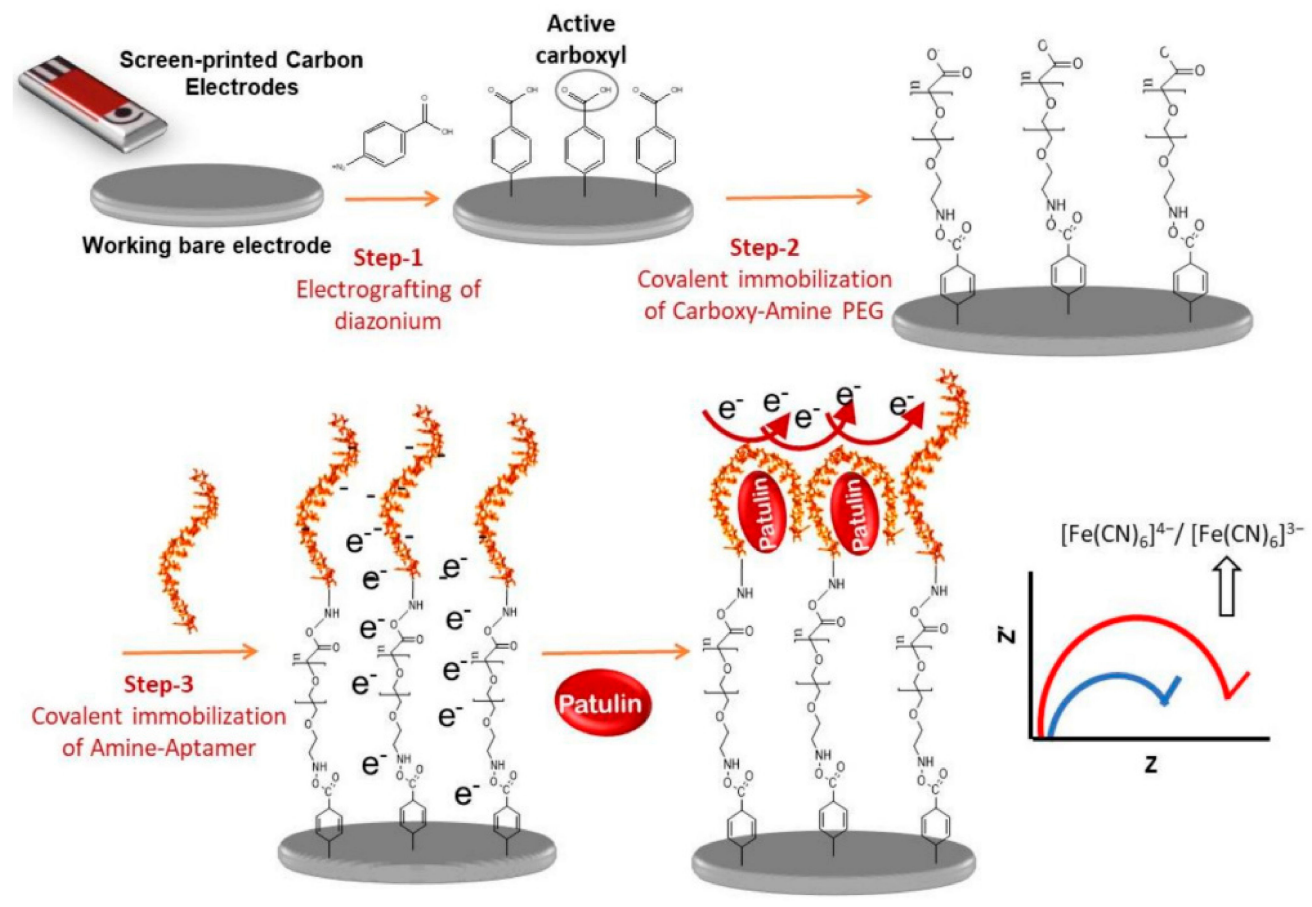
Design and working principle of an impedimetric aptasensor for patulin employing a detection platform obtained via diazonium electrografting. Reprinted from [76].

Numerous aptamer-based assays employing an aptamer sequence developed by Wu et al. through GO-SELEX screening [77] have been adopted for PAT detection in apple juice samples [76,77,233,234]. A carboxyphenyl-functionalized electrochemically grafted SPCE platform was also exploited by Marty’s group for patulin aptasensors [76]. Long linear carboxyl-amino bifunctional PEG molecules were coupled through amide bonds at the carboxyl groups of the grafted aryl layer, which enabled the subsequent binding of the amino-modified aptamer (Figure 8). According to these authors, the immobilized PEG chains acted like “tunnels” for the redox probes’ charge transfer, while aptamers served as “gates” for these tunnels. The conformational changes of the aptamer in the presence of the target regulate the closing of the “gates” and consequently induce an increase in charge transfer resistance (measured by EIS) for the redox species in the solution. The response was linear in the 1–25 ng L^−1^ concentration domain, with an LOD of 2.8 ng L^−1^. Moreover, the sensor showed a selective response in the presence of mycotoxins commonly found in food, and its suitability for the analysis of real samples was demonstrated using fresh apple juice spiked with different concentrations of PAT (recoveries of 92.5–96%, ±4.8%) [76].

### 5.4. Electrochemical Biosensors for Lysozyme

Lysozyme (LYS) is a relatively small-sized enzyme (EC 3.2.1.17), also known as muramidase or N-acetylmuramide glycanhydrolase. This enzyme is ubiquitous in nature, being found in almost all body fluids, animal tissues, plants, and bacteria [235]. LYS attacks the polysaccharide constituents of lactic acid bacteria cell walls and thus exhibits selective antimicrobial activity [235]. Lysozyme extracted from chicken egg white is considered a natural antimicrobial agent and in several countries it qualifies as a food preservative for cheese, beer, shrimps, and sausages [79]. LYS is also applied in the process of wine manufacturing for suppressing heterolactic fermentation [78], and the Organization of Vine and Wine recommends a maximum concentration of 500 mg L^−1^ LYS for the purpose of wine aging [78,236]. The EU food safety agency found that egg-derived LYS may trigger adverse allergic reactions in susceptible individuals after moderate wine consumption [237].

Marty’s group developed two strategies for LYS detection, exploiting a detection platform based on SPCEs modified with carboxyphenyl functionalities through diazonium electrografting [78,79]. The first strategy was similar to the one employed by the same group for the detection of other foodborne contaminants, which was described above [68,70]. A comparison of biosensors employing two different aptamers (designated as COX and TRAN, after the names of researchers who contributed to their selection [80,83]) immobilized through carbodiimide coupling on carboxyphenyl grafted layers was provided [78]. The LYS detection was achieved through EIS by monitoring the increase in R_ct_ of the [Fe(CN)_6_]^4−/3−^ redox probe due to the blocking and electrostatic repulsion of negatively charged oligonucleotides immobilized at the electrode. Further treatment steps, i.e., incubation in ethanolamine to block the unreacted carboxyl groups and in BSA to suppress nonspecific adsorption, induced a decrease and then an increase of R_ct_, respectively. As the addition of LYS resulted in a further increase in R_ct_, despite its iso-electric point, which is around 11, the authors concluded that steric effects dominate over the electrostatic attraction. Moreover, for the TRAN-based aptasensor the increase of R_ct_ was higher than for COX, resulting in a higher sensitivity. Although EIS results were fully consistent with CV studies, the EIS method displayed a better sensitivity. The authors reported good linearity domains and LODs, 0.1–0.8 μM and 100 nM using COX and 0.025–0.8 μM and 25 nM using TRAN, respectively. Moreover, good selectivity, stability, and reproducibility for LYS detection were obtained in both cases, and the reliability and suitability of the assay for real wine samples was tested by spiking wine aliquots with different concentrations of LYS (recoveries in the range from 96.4 to 102%) [78]. A very similar concept for the development of an aptasensor for wine analysis was later adopted by Ortiz-Aguayo and del Valle, who chose graphite–epoxy composite electrodes as substrates [81]. The second approach for LYS detection proposed by Marty’s group was an aptamer–antibody sandwich assay that employed the same aptamer immobilization method consisting of carbodiimide-mediated coupling at an -COOH functionalized interface [79]. After the aptamer–target binding, a secondary antibody containing biotin functionalities was used to form a sandwich structure, enabling the interaction with an avidin-modified ALP enzyme label. The analytical signal was generated in the presence of 1-naphthyl phosphate as the ALP substrate, which is hydrolyzed to electroactive 1-naphthol. The 1-naphthol oxidation current, registered using DPV, varied linearly with the LYS concentration in the range from 5 fmol L^−1^ to 5 nmol L^−1^, with an LOD of 4.3 fmol L^−1^ [79]. Several control experiments carried out in the presence of BSA, cytochrome c, and casein indicated the good specificity of the assay. Moreover, the authors proved that this biosensor had good stability and reproducibility and could be successfully applied for detecting LYS in spiked wine samples (recovery rates of 95.2–102.0%) [79]. The comparison of the two strategies for LYS detection presented above clearly indicates higher performance for the aptamer–antibody sandwich assay in terms of the LOD and concentration range.

Another aptasensor for LYS was developed by Szunerits and co-workers for clinical applications and tested on serum samples from patients suffering from inflammatory bowel disease. The detection platform consisted of vertically aligned nitrogen-doped carbon nanotubes (VA-NCNTs) electrochemically grafted with carboxyphenyl groups in order to allow aptamer coupling, thus achieving femtomolar detection of LYS in serum and urine without any additional amplification [82]. A two-step aptamer coupling strategy was adopted: neutravidin was first attached to carboxyl-terminated VA-NCNTs through amide bonds, followed by functionalization with biotinylated aptamer through neutravidin–biotin interactions. The sensor response was evaluated through the change in the DPV oxidation current of [Fe(CN)_6_]^4−^ upon analyte addition, which induced conformational changes in the aptamer molecule, and partially hindered electron transfer between the redox probe and electrode. A similar sensor based on VA-CNTs (without nitrogen doping) displayed a linear response for a similar concentration range (0.1–7 pM), but the sensitivity was more than two times lower. The selectivity of the assay toward LYS was associated primarily with the covalent integration of the LYS aptamer at the nanointerface, while its sensitivity was attributed to the fast heterogeneous electron transfer at the VA-NCNTs platform [82].

Another approach for developing sensitive amperometric aptasensors for LYS employed RGO/PEI composites obtained through electrophoretic deposition (EPD), which were functionalized with aryl groups bearing alkynyl substituents and subsequently coupled with azide-modified LYS aptamers through a “click” reaction [84]. The EPD conditions employed for electrode fabrication allowed the simultaneous reduction of GO to RGO and of aryldiazonium cations to aryl radicals. The detection process relied on the increase in the DPV oxidation current of [Fe(CN)_6_]^4−/3−^ due to aptamer conformational changes upon analyte binding. The binding event hinders the negatively charged phosphate groups on the oligonucleotide chains, facilitating the interaction between the negatively charged probe and the positive charges on the RGO/PEI matrix [84]. The aptasensor responded linearly up to 30 pM LYS, with an LOD of 200 fM. Obvious advantages of this strategy are the one-step fabrication of the detection platform, which is very convenient for large scale production, and the improved assay sensitivity and LOD associated with the synergic combination of the large surface area and enhanced electron transfer properties of RGO/PEI-modified electrodes [84].

### 5.5. Electrochemical Biosensors for Cadmium Ions

Cadmium is one of the most toxic metals for humans, causing many serious diseases, but it is widely utilized in fertilizers, plastics, and other industrial products [87,238]. Cadmium is relatively poorly absorbed into the body, yet it accumulates in the kidneys when adsorbed, and causes renal damage. Kidneys in feedstock represent a major dietary source of Cd^2+^, although lower levels are also found in many other foods, such as cereals, fruit, vegetables, meat, and fish [239]. Concentrations of cadmium are in the range of 1–50 µg kg^−1^ in meat, fish, and fruit and 10–300 µg kg^−1^ in wheat, rice, and potatoes, while the highest levels of 100–1000 µg kg^−1^ are found in the kidneys and livers of mammals and in several species of mussels [240].

An aptasensor for cadmium detection in water, involving diazonium electrochemistry for aptamer immobilization at a gold substrate electrode, has been reported very recently [86]. The strategy was similar to the one employed in the previously reported aptasensing assay for food contaminants, with aptamer sequences coupled via carbodiimide chemistry at the carboxyphenyl functionalities of the grafted aryl layer. The EIS technique was used for Cd^2+^ detection, and the sensor response was recorded by monitoring the R_ct_ increase for the [Fe(CN)_6_]^4−/3−^ couple due to the conformational changes induced by aptamer switching from a random coil structure to an aptamer–target complex [86]. The authors reported a linear relationship between the R_ct_ and the logarithm of Cd^2+^ concentration in the 10^−3^–10^−9^ mol L^−1^ domain, with an LOD of 2.75 × 10^−10^ mol L^−1^. The sensor also displayed a high selectivity for Cd^2+^ in the presence of interfering cations, such as Pb^2+^ and Zn^2+^. A slight cross-reactivity was noted for Hg^2+^, which was attributed to the formation of special T–Hg–T mismatched base pairs [86,241]. By applying the standard addition method, the suitability of this sensing assay for cadmium in spring water was successfully demonstrated, and the results were validated by atomic absorption spectroscopy, a certified method for cadmium detection (90.12% recovery rate) [86].

### 5.6. Electrochemical Aptasensors for Bacteria

As bacteria are a significant cause of foodborne illness worldwide [89], the development of simple and sensitive techniques that can detect and discriminate bacterial pathogens in real-time may have significant implications for the management of bacterial infections, especially in clinical diagnosis [242]. The most common foodborne pathogen bacteria detected using aptasensors are E. coli [89], S. aureus [209], S. typhimurium [15,88], and L. monocytogenes [243]. Salmonellae are important bacterial pathogens in food animal species [89]. Amongst more than 2500 serotypes of Salmonella, a prevalent serotype responsible for about a third of worldwide cases of foodborne diseases is Salmonella enterica serovar Typhimurium (S. Typhimurium) [15,88].

Beni’s group reported the fabrication of a label-free impedimetric aptasensor for S. Typhimurium, with SPCEs modified through diazonium chemistry as transducer electrodes [88]. The aptamer sequence used in this study was designed by Joshi et al. to complement an outer membrane protein found in S. Typhimurium as specific binding target [89]. The authors compared the electrochemical grafting method and Zn-mediated chemical grafting in terms of the surface density of the aptamer layer and sensitivity. The electrochemical grafting of carboxyphenyl using the corresponding diazonium salt promoted the formation of a denser aptamer biorecognition layer than the Zn-mediated method. For quantifying the aptamer surface density, they applied the well-known chronocoulometric procedure based on the proportionality between the number of phosphate residues from oligonucleotide strands and the amount of the charge-compensating RuHex marker [116]. The aptasensor fabricated by employing the electrochemical route proved to be more sensitive at both low (1 × 10^2^ CFU mL^−1^) and high (1 × 10^8^ CFU mL^−1^) concentrations of S. typhimurium, in comparison with the device obtained by Zn-mediated chemical grafting. The aptasensor displayed a linearity domain in the 1 × 10^1^–1 × 10^8^ CFU mL^−1^ concentration range, with an LOD of 6 CFU mL^−1^, and was highly selective for S. typhimurium. Moreover, the study also reported a facile regeneration of the sensor, the dissociation of the aptamers from the bacteria after the sensor immersion in 2 M NaCl solution for 30 min being demonstrated by impedance and staining experiments. The recovery studies on spiked apple juice samples demonstrated the applicability of this approach for real sample analysis. Also, an important advantage of the assay over previous work proved to be its suitability for detecting S. typhimurium in undiluted juice samples [88].

Another approach involving diazonium chemistry was employed by Lian et al. for the fabrication of a piezoelectric sensor for S. aureus [209]. The inclusion of graphene as transducer element in the aptasensor design greatly improved the device sensitivity. These authors used a mercaptobenzene diazonium salt as a molecular bridge to connect the interdigitated gold electrodes (IDE) and graphene and then aptamers were immobilized on the graphene surface via the π–π stacking of nucleotide bases. The detection was carried out by monitoring the change in the oscillation frequency of the piezoelectric quartz crystal used as substrate for the IDE electrodes in the concentration range from 4.1 × 10 to 4.1 × 10^5^ CFU mL^−1^, with an LOD of 41 CFU mL^−1^. The aptasensor could be regenerated by thoroughly washing the electrode with acetonitrile and acetone, followed by incubation in an aptamer solution at 37 °C for 16 h. The authors reported a decrease in the frequency shift by less than 5% after 10 regenerations. The method proved also to be specific for S. aureus and its feasibility for milk analysis was demonstrated by testing milk samples inoculated with different concentrations of S. aureus. The validation of results was performed using the classical plate-counting method [209].

## 6. Conclusions

The broad impact of food contaminants on everyday life requires reliable and efficient detection methods. Although the complexity of food matrices and the trace levels at which food contaminants are commonly encountered makes this a challenging task, biosensors are quickly becoming effective analytical devices for the fast screening of food contaminants [244]. The use of aptamers as recognition elements brings further advances in detection performance, as they exhibit high binding affinities for their target analytes, enhanced stability, and lower fabrication costs.

A multitude of reviews have been published lately describing the progress in aptasensors intended for food quality and safety monitoring [2,16,42,190,243,245,246,247,248,249,250]. This paper brings a new perspective on this subject by emphasizing the contribution of aryldiazonium chemistry in developing aptasensing assays for commonly encountered food contaminants. The particular focus on electrochemical grafting emerged from its ability to promote the proper surface density, distribution, structural flexibility, and stability for biorecognition elements necessary for a successful detection process. The stability of biological receptors immobilized within the sensing interface through aryl diazonium grafting is an important issue for real sample analysis conditions and constitutes an important criterion for large scale biosensor commercialization [3]. Many studies completed so far revealed the significant influence of the flexibility and surface density of biorecognition molecules on the sensitivity of electrochemical aptasensors [14,19,93]. Since the electrografting of protected aryldiazonium salts has demonstrated its effectiveness for the preparation of monolayers with controlled structure, the use of grafted aryl groups as anchors for aptamers is an effective tool for the adjustment of the surface density of biorecognition molecules. Moreover, diazonium grafted layers allow the immobilization onto different substrates of adequately spaced oligonucleotide probes, which improves the efficiency of the recognition process and sensor stability over time. The electrografting of a binary layer of nitrophenyl and phenylethynyl groups proved to be a simple way to suppress the non-specific response from the sample matrix and to increase the assay sensitivity [40]. Several recent studies showed that the inclusion into the biorecognition layer of aryl groups bearing ethylene glycol or various zwitterionic groups improves the selectivity of aptasensing devices by ensuring an efficient antifouling resistance for proteins, DNA, or other molecules [35,195].

This review provides a comprehensive summary of electrochemical aptasensors fabricated via aryldiazonium grafting, classified and discussed according to the type of food contaminant. The aptasensors considered here displayed LODs ranging from the femtomolar to the micromolar level and proved their effectiveness for detecting trace amounts of food contaminants in accordance with concentration limits set by specific legislation and with analytical characteristics competitive with established analytical methods. Only a limited number of studies investigated the regeneration of the sensing surfaces [40,88,209], although it should represent an important characteristic in the design of aptasensors.

Despite the remarkable performances achieved by aptasensors in terms of their ability to detect low levels of target analytes, numerous issues must still be addressed for their further development. The process of selecting a specific aptamer sequence is a tedious task and, in spite of the intensified research efforts in the last few years, the number of published aptamer sequences is still limited and corresponds to a narrow range of analytes [3,229]. Taking advantage of the latest advances in computational methods and in silico design, the rational and efficient design of aptamers with improved target affinity and selectivity will soon be possible [245]. Another key aspect is the development of platforms capable of multiplex analysis, without compromising the required accuracy and specificity [251]. Several important advancements have already been made towards the fabrication of such “universal toolsets”, enabled by diazonium functionalization strategies [38,193]. In this regard, the covalent surface modification of transducer electrodes through diazonium chemistry proved useful for obtaining mixed monolayers of controlled composition, enabling future developments in multiplexed recognition [36].

Future advances in this field are also expected from a commercial perspective. Although the use of electrochemical aptasensors for food contaminants presents clear advantages over traditional methods, very few are currently available on the market. Alongside validation by regulatory authorities, improving the performance characteristics and costs will undoubtedly contribute to the commercial success of electrochemical aptasensing assays intended for food safety monitoring [251]. Thus, further in-depth studies should aim at optimizing immobilization protocols and extending the use of nanomaterials in aptasensor design, in order to boost the analytical performance of current devices and lead to the general acceptance of this type of assay [252,253].

## Figures and Tables

**Table 1:** Aptamer sequences obtained through SELEX technology and employed in the fabrication of electrochemical aptasensors for food contaminants.

Detected Analyte	Reference (Aptasensor)	Aptamer Sequence	Kd	Reference (SELEX Aptamer)
TET	[25,59]	5′- CGT ACG GAA TTC GCT AGC CCC CCG GCA GGC CAC GGC TTG GGT TGG TCC CAC TGC GCG TGG ATC CGA GCT CCA CGT G-3′	63 nM	[60]
OTC	[61]	5′-GGA ATT CGC TAG CAC GTT GAC GCT GGT GCC CGG TTG TGG TGC GAG TGT TGT GTG GAT CCG AGC TCC ACG TG-3′	11.13 nM	[62]
Kan	[63]	5′-TGG GGG TTG AGG CTA AGC CGA-3′	78.8 nM	[64]
Pen-G	[65]	5′-GGG AGG ACG AAG CGG AAC GAG ATG TAG ATG AGG CTC GAT CCG AAT GCG TGA CGT CTA TCG GAA TAC TCG TTT TTA CGC CTC AGA AGA CAC GCC CGA CA-3′	-	[65]
E2	[66]	5′-GCT TCC AGC TTA TTG AAT TAC ACG CAG AGG GTA GCG GCT CTG CGC ATT CAA TTG CTG CGC GCT GAA GCG CGG AAG C-3′ (truncated to 38 mer)	2.7 nM	[67]
OTA	[40,68,69,70]	5′-GAT CGG GTG TGG GTG GCG TAA AGG GAG CAT CGG ACA-3′	0.2 μM	[71]
AFB1	[72]	5′-GTT GGG CAC GTG TTG TCT CTC TGT GTC TCG TGC CCT TCG CTA GGC CCA CA-3′	75 nM	[73]
AFM1	[74]	5′-ACT GCT AGA GAT TTT CCA CAT-3′	-	[75]
Pat	[76]	5′-GGC CCG CCA ACC CGC ATC ATC TAC ACT GAT ATT TTA CCT T-3′	21.83 nM	[77]
LYS	[78,79]	5′-NH2-GCA GCT AAG CAG GCG GCT CAC AAA ACC ATT CGC ATG CGG C-3′	2.8 nM	[80]
LYS	[78,81,82]	5′-NH2-ATC AGG GCT AAA GAG TGC AGA GTT ACT TAG-3′	31 nM	[83]
LYS	[84]	5′-GGG AAT GCA TCC ACA TCT ACG AAT TCA TCA GGG CTA AAG AG-3′	29 ± 5 nM	[85]
Cd^2+^	[86]	5′-ACC GAC CGT GCT GGA CTC TGG ACT GTT GTG GTA TTA TTT TTG GTT GTG CAG TAT GAG CGA GCG TTG CG-3′	34.5 nM	[87]
S. typhimurium	[88]	5′-TTT GGT CCT TGT CTT ATG TCC AGA ATG CGA GGA AAG TCT ATA GCA GAG GAG ATG TGT GAA CCG AGT AAA TTT CTC CTA CTG GGA TAG GTG GAT TAT-3′	-	[89]

**Table 2:** Analytical performance of electrochemical aptasensors for food contaminants developed using diazonium chemistry.

Analyte	Substrate	Diazonium Salt/Antifouling Layer; Apt Coupling Method	Apt Selection Reference	Detection Method; Principle	LOD; Linearity Range	Real Sample; Recovery Rate	Reference
TET	SPCE	4-carboxyphenyl; EDC-NHS; 5′-amino-Apt	[60]	CV; Apt intrinsic redox activity	0.035 μg L^−1^; 0.05 μg L^−1^–20 μg L^−1^	Natural waters; 87.8–97%	[25]
OTC	GC	4-carboxyphenyl/ BSA; EDC-NHS; 5′-amino-Apt	[62]	DPV; [Fe(CN)_6_]^3−/4−^ redox probe	0.229 μg L^−1^; 1.0 μg L^−1^–1.0 × 10^5^ μg L^−1^	Spiked milk; 87.0–99.3%	[61]
TET	SPCE	4-carboxyphenyl; EDC-NHS; 5′-amino-Apt	[60]	SECM; Apt intrinsic redox activity	-	-	[59]
Kan	SPCE	4-carboxyphenyl/Casein; EDC-NHS; 3′-amino-Apt	[64]	EIS; [Fe(CN)_6_]^3−/4−^ redox probe	0.11 μg L^−1^; 1.2 μg L^−1^–75 μg L^−1^	Spiked milk; 96.88–100.5%	[63]
Pen-G	SPCE	4-nitrophenyl, electrochemically reduced to 4-aminophenyl/Casein; GA crosslinking; 5′-amino-Apt	[65]	EIS; [Fe(CN)_6_]^3−/4−^ redox probe	0.17 µg L^−1^; 0.4–1000 µg L^−1^	Spiked milk; 83–100%	[65]
E2	ERGO/GC	4-carboxyphenyl; EDC-NHS; 5′-amino- Apt	[67]	SWV; E2 redox activity	0.5 × 10^−15^ mol L^−1^; 1.0 10^−15^ mol L^−1^–9.0 10^−12^ mol L^−1^ and 1.2 10^−11^ mol L^−1^–2.3 × 10^−10^ mol L^−1^	Waste waters: 94.05–100.47%; pharmaceutical samples: 96.87%–100.22%	[66]
OTA	SPCE	4-ethynyl phenyl (protected), 4-nitrophenyl; “click” chemistry; 5′-azido- Apt	[71]	EIS; [Fe(CN)_6_]^3−/4−^ redox probe	0.25 μg L^−1^; 1.25 μg L^−1–^500 μg L^−1^	Spiked beer samples	[40]
OTA	SPCE	4-carboxyphenyl/ BSA; EDC-NHS; 5′-amino-Apt	[71]	EIS; [Fe(CN)_6_]^3−/4−^ redox probe	0.15 μg L^−1^; 0.15–2.5 μg L^−1^	Spiked cocoa samples; 91–95%	[68]
OTA	SPCE	4-carboxyphenyl/ BSA; EDC-NHS; 5′-amino-Apt	[71]	DPV; oxidation current of 1-naphthol (ALP enzyme, 1-NPP substrate)	0.07 μg L^−1^; 0.15–5 μg L^−1^	Spiked cocoa samples; 82.1–85%	[69]
OTA	PGE	4-carboxyphenyl; EDC-NHS; 5′-amino- Apt	[71]	EIS; [Fe(CN)_6_]^3−/4−^ redox probe	0.1 μg L^−1^; 0.1–2 μg L^−1^	Spiked beer samples; 93.4 ± 6.6%	[70]
AFB1	SPCE	4-carboxyphenyl/ BSA; EDC-NHS; 5′-amino- Apt	[73] (seq B)	EIS; [Fe(CN)_6_]^3−/4−^ redox probe	Seq. A: 0.125 μg L^−1^; 0.25–16.0 μg L^−1^ and Seq. B: 0.25 μg L^−1^; 0.1–16.0 μg L^−1^	Spiked beer and wine samples; 92–102%	[72]
AFM1	GCE	4-carboxyphenyl; EDC-NHS; -5′-amino- Apt or 5′-HEG-amino-Apt	[75]	EIS; [Fe(CN)_6_]^3−/4−^ redox probe	1.15 ng L^−1^, 0.02 μg L^−1–^1 μg L^−1^(for 5′-HEG-amino-Apt)	Spiked milk; 98%–114%	[74]
PAT	SPCE	4-carboxyphenyl/ BSA; EDC-NHS + carboxy-amino PEG; 3′-amino-Apt	[77]	EIS; [Fe(CN)_6_]^3−/4−^ redox probe	2.8 ng L^−1^; 1–25 ng L^−1^	Spiked fresh apple juice; up to 99%	[76]
LYS	SPCE	4-carboxyphenyl/ BSA; EDC-NHS; 5′-amino-Apt	[80,83]	EIS; [Fe(CN)_6_]^3−/4−^ redox probe	25 nM; 0.025–0.8 μM for Apt [80] and 100 nM; 0.1–0.8. μM for Apt [83]	Spiked wine; 94.2–102%	[78]
LYS	GECE	4-carboxyphenyl/ PEG; EDC-NHS; 5′-amino-C_6_-Apt	[83]	EIS; [Fe(CN)_6_]^3−/4−^ redox probe	1.67 µM; 1.67 µM–5 µM	Spiked wine; 77%	[81]
LYS	SPCE	4-carboxyphenyl/ BSA; EDC-NHS; 5′-amino-Apt + LYS-Ab-Bio/Av-ALP (sandwich assay)	[80]	DPV; oxidation current of 1-naphthol (ALP enzyme, 1-NPP substrate)	4.3 fmol L^−1^; 5 fmol L^−1^–5 nmol L^−1^	Spiked wine; 95.2–102.0%	[79]
LYS	VA-NCNT	4-carboxyphenyl; EDC-NHS + NeutrAv; 5′-Bio-T_24_-Apt	[83]	DPV; [Fe(CN)_6_]^4−^ oxidation signal	100 fM; 0.1–7 pM	Serum samples	[82]
LYS	RGO/PEI	4-ethynylphenyl/ BSA; “click” chemistry; 5′-azido-Apt	[85]	DPV; [Fe(CN)_6_]^4−^ oxidation signal	1 pM; 1–15 pM	Serum samples	[84]
Cadmium	Au SPE	4-carboxyphenyl; EDC-NHS; 5′-amino-Apt	[87]	EIS; [Fe(CN)_6_]^3−/4−^ redox probe	2.75 × 10^−10^ mol L^−1^; 10^−3^–10^−9^ mol L^−1^	Real water samples	[86]
S. typhimurium		4-carboxyphenyl; EDC-NHS; 5′-amino-Apt	[89]	EIS; [Fe(CN)_6_]^3−/4−^ redox probe	6 CFU mL^−1^; 1 × 10^1^–1 × 10^8^ CFU mL^−1^	Spiked apple juice	[88]

SPCE: screen-printed carbon electrode; Apt: aptamer; GC: glassy carbon electrode; GA: glutaraldehyde; ALP: alkaline phosphatase; 1-NPP: 1-naphthyl phosphate; PGE: pencil graphite electrodes; HEG: hexaethyleneglycol; GECE: graphite–epoxy composite electrodes; Ab: antibody; Bio: biotin; Av: avidin; NeutrAv: neutravidin; T: timine; RGO: reduced graphene oxide; PEI: polyethyleneimine; CFU: colony-forming unit.
